# Supplementary figures and images for: New genomic techniques, old divides: Stakeholder attitudes towards new biotechnology regulation in the EU and UK
Source: PLoS One. 2024 Mar 6;19(3):e0287276. doi: 10.1371/journal.pone.0287276 (PMC10917245; doi:10.1371/journal.pone.0287276)

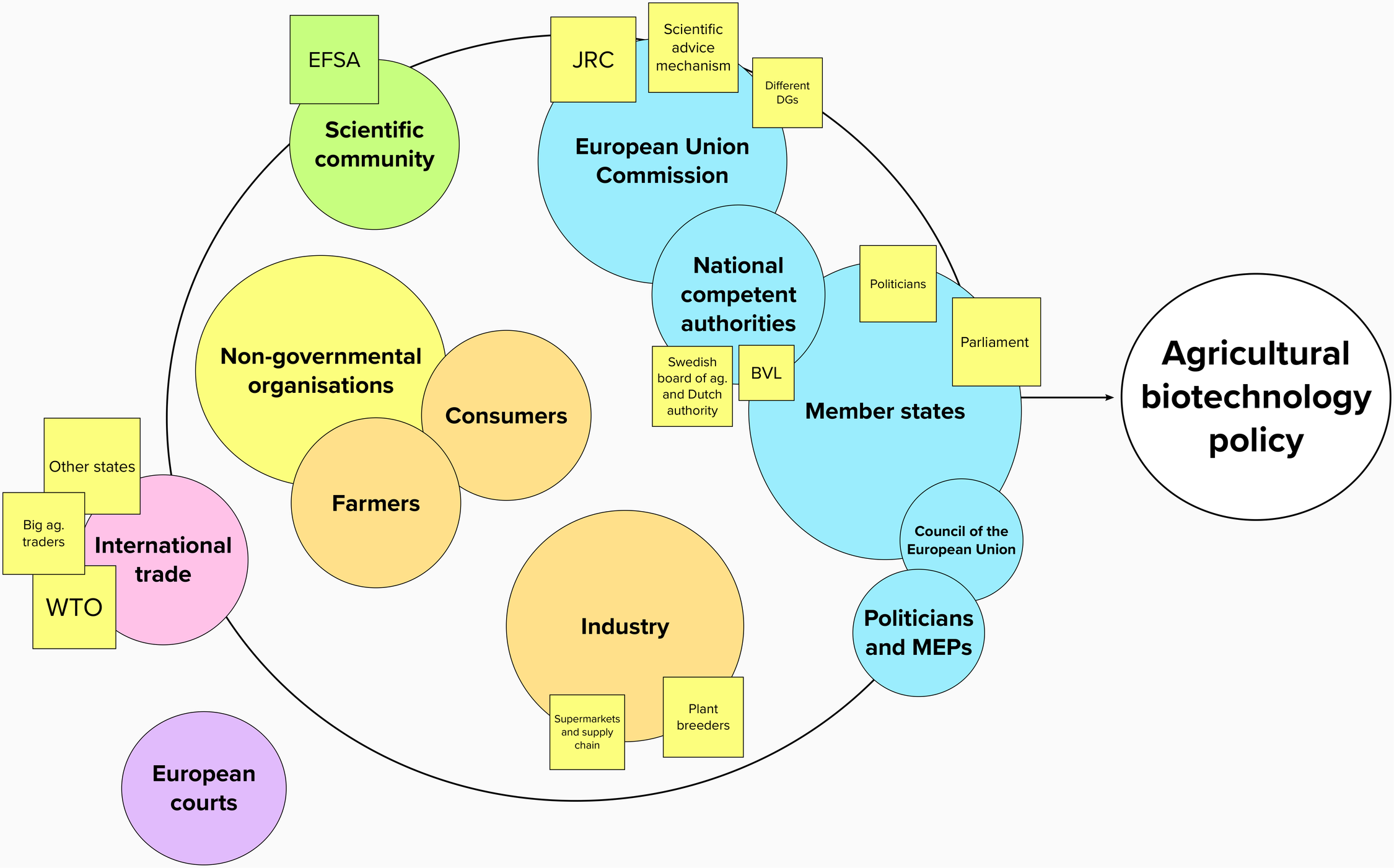

Supplement: S1 Fig — This is the result of the mapping exercise undertaken with participants in the European Union. Size of circles does not indicate importance. (TIF) [file pone.0287276.s001.tif]
