# Supplementary material for: New genomic techniques, old divides: Stakeholder attitudes towards new biotechnology regulation in the EU and UK
Source: PLoS One. 2024 Mar 6;19(3):e0287276. doi: 10.1371/journal.pone.0287276 (PMC10917245; doi:10.1371/journal.pone.0287276)
Supplement: S1 File — Codebook with list of codes used in the analysis of this research. (DOCX) [file pone.0287276.s002.docx]

New Genomic Techniques, Old Divides: stakeholder attitudes towards new biotechnology regulation in the EU and UK - Codebook

Codes

| Name | Description | Files | References |
| --- | --- | --- | --- |
| Biological lines in the sand | This node codes for the arguments participants made around the nature of different forms of biotechnology that inform their attitudes towards regulation. | 11 | 38 |
| Definitions of biotechnology |  | 3 | 5 |
| Genetic technologies aren't the problem |  | 2 | 2 |
| Plants easier than animals |  | 1 | 1 |
| Challenges | This node codes for challenges for the European agri-food system. | 7 | 12 |
| Considerations for regulation | This node codes for attitudes towards the regulation of biotechnology in Europe. | 15 | 119 |
| 2001.18.EC |  | 5 | 11 |
| Association members have different levels of interest in NGTs |  | 1 | 2 |
| Backyard gene editing |  | 1 | 1 |
| Building trust in institutions |  | 1 | 1 |
| Clarity is imperative |  | 1 | 1 |
| Co-existence and contamination |  | 3 | 6 |
| Definitions of what is natural are legally suspect |  | 1 | 3 |
| Detection |  | 3 | 8 |
| Developers add more than just one gene |  | 2 | 2 |
| Ethics |  | 1 | 1 |
| EU-UK contrast |  | 6 | 10 |
| Existing regulations reflect disapproval |  | 1 | 1 |
| Extensive governance |  | 1 | 1 |
| Foot in the door |  | 1 | 1 |
| Harmonisation |  | 1 | 2 |
| It's 'just a tool' |  | 1 | 3 |
| Labelling and traceability |  | 5 | 6 |
| Little difference between GMOs and NGTs |  | 2 | 2 |
| NGTs prompting action for NGOs |  | 1 | 1 |
| Patenting and IP |  | 4 | 11 |
| Public good |  | 1 | 3 |
| Pushing the Commission |  | 2 | 2 |
| Risk assessment |  | 11 | 28 |
| Process-based assessment |  | 3 | 3 |
| Product versus process |  | 3 | 4 |
| Product-based assessment |  | 4 | 5 |
| Provisions are already there... |  | 3 | 4 |
| Rush or push for de-regulation |  | 2 | 3 |
| Things have changed since 2001 |  | 3 | 3 |
| UK-wide regulation rather than devolved |  | 1 | 1 |
| Withdrawal of chemistry not matched by approval of genetics |  | 1 | 1 |
| Key actors and pathways to influence | This node codes for the opinions of participants towards different actors within the agri-food system and their relative influence. It also codes for details on how different actors attempt to influence biotechnology policymaking. | 14 | 45 |
| Better to trust experts |  | 1 | 1 |
| Big companies and corporate control |  | 6 | 17 |
| Bringing together knowledge and people |  | 7 | 12 |
| Connections with politicians is crucial |  | 4 | 8 |
| Consultations and copy-paste opinions |  | 14 | 40 |
| Difficult to know how to influence policy |  | 1 | 1 |
| Disruptive business models |  | 1 | 1 |
| Influence of supermarkets |  | 3 | 3 |
| Lobbying |  | 6 | 7 |
| Move beyond 'stakes' |  | 1 | 1 |
| Need to work with farmers early in technology development |  | 1 | 1 |
| Not possible to chat anymore |  | 1 | 1 |
| Opinions on different actors |  | 15 | 77 |
| Commission |  | 4 | 6 |
| Farmers |  | 1 | 1 |
| Frustration |  | 1 | 1 |
| Government bodies |  | 1 | 3 |
| Industry |  | 5 | 7 |
| Journalists |  | 1 | 2 |
| Member States |  | 4 | 5 |
| NGOs |  | 11 | 16 |
| Politicians |  | 3 | 3 |
| Public |  | 5 | 9 |
| Scientists |  | 5 | 10 |
| Slow Commission, short Parliament | This node codes for data relating to slow or ineffective Commission regulatory processes and the shortness of Parliamentary terms that hamstring regulatory progress. | 3 | 10 |
| Supermarkets |  | 1 | 1 |
| The public doesn't understand |  | 1 | 1 |
| Public engagement |  | 3 | 4 |
| Relative influence of different actors evolves over time |  | 1 | 2 |
| Scientific reporting |  | 2 | 2 |
| Personal history | This node codes for participants’ personal histories and isn’t included in the coded data associated with this publication. | 15 | 35 |
| Risks and benefits of biotechnology | This node codes for anything related to the perceived risks and benefits of biotechnology. | 14 | 110 |
| (Broken) promises and non-delivery |  | 6 | 14 |
| Analysis of risks and benefits |  | 1 | 1 |
| Benefits |  | 8 | 15 |
| Biological contamination |  | 1 | 1 |
| Biotechnology furthers monoculture and corporate control |  | 1 | 1 |
| Cisgenesis good, transgenics bad |  | 5 | 5 |
| Conventional breeding |  | 1 | 2 |
| Debate more nuanced in medical science |  | 1 | 1 |
| Easy to cast aspersions, hard to convince otherwise |  | 1 | 1 |
| EU wants transition, NGTS can be part of it |  | 1 | 1 |
| Europe falling behind |  | 3 | 4 |
| Forming an opinion |  | 1 | 1 |
| Impacts of non-adoption |  | 2 | 5 |
| Improving on nature |  | 2 | 3 |
| Intended users of biotechnology |  | 1 | 2 |
| Little middle ground |  | 1 | 1 |
| Misunderstanding of opponents' arguments |  | 1 | 1 |
| Need to focus on other things |  | 5 | 14 |
| Alternatives to NGTs |  | 2 | 6 |
| Importance of social and ecological innovation |  | 1 | 1 |
| Technology can be overhyped |  | 1 | 2 |
| No struggle, no need for technology |  | 2 | 2 |
| Parallels between NGTs and vaccines |  | 3 | 5 |
| Plants are complex |  | 2 | 4 |
| Popular depictions of biotechnology not accurate |  | 1 | 1 |
| Risks are being simplified |  | 1 | 2 |
| Shared values |  | 1 | 2 |
| The issues are well known |  | 1 | 1 |
| Unintended consequences, knock-on effects and unknown unknowns |  | 5 | 17 |
| Unknown health impacts |  | 2 | 2 |
| What is the vision for gene editing |  | 1 | 1 |
