# Supplementary material for: New genomic techniques, old divides: Stakeholder attitudes towards new biotechnology regulation in the EU and UK
Source: PLoS One. 2024 Mar 6;19(3):e0287276. doi: 10.1371/journal.pone.0287276 (PMC10917245; doi:10.1371/journal.pone.0287276)
Supplement: S2 File — Data coded to each code used in the analysis of this research. (DOCX) [file pone.0287276.s003.docx]

**Name:** Biological lines in the sand

**<Files\\Ambemore> - § 4 references coded [2.16% Coverage]**

**Reference 1 - 0.22% Coverage**

… cisgenesis it’s maybe much more GMO because you use an external DNA…

**Reference 2 - 0.80% Coverage**

If we look only at the techniques that deliver a product that is similar to a conventional we believe that we do not need any kind or risk assessment because in fact in the end, the product you cannot distinguish from the conventional varieties…

**Reference 3 - 0.80% Coverage**

… if we consider it as a GMO to some extent you need to adapt the risk assessment if you cannot make a distinction but if you have used in between the process an external DNA to make your modification, yes you need to some extent risk assessment.

**Reference 4 - 0.33% Coverage**

But we believe if it is mutagenesis this is not necessary because we have not used an external DNA.

**<Files\\Illabong> - § 1 reference coded [1.61% Coverage]**

**Reference 1 - 1.61% Coverage**

No, one thing that we figured out is that if it is not going into food, then that seems to be acceptable. So bio-based products such as making recyclable packaging material etc., this seems to be at least we feel where our consumers like those products more than the conventional ones.

**<Files\\Jata> - § 1 reference coded [1.72% Coverage]**

**Reference 1 - 1.72% Coverage**

Well, the standpoint of the scientist is the following so, whatever crop that we can obtain with genome editing that is non-distinguishable from the plant that is obtained by traditional breeding, should be regulated the same so, we call it traditional like new genomic techniques products or plants should be regulated exactly the same as traditionally bred plants.

**<Files\\Kebo> - § 2 references coded [5.83% Coverage]**

**Reference 1 - 3.61% Coverage**

So for example with gene editing, something people often ignore is the fact that when you do gene editing or when most people do gene editing they make a transgenic plant that carries an inserted DNA that expresses Cas9 and guide RNAs and then they go and hit something and change it and then you cross out the Cas9. Now when you do use the GM method like for gene editing or for using the GM method, basically what happens is the agrobacterium, most people use agrobacterium, it pumps some DNA into the plant and some of it gets incorporated and is expressed but sometimes bits of DNA go in elsewhere, compared to the scale of genetic differences between let’s say inbred lines of maize, any change due to that is tiny compared to the enormous amount of natural variation but anyway, I think people can be entitled to ask have these guys checked if there’s any other DNA in there, have they really got rid of the Cas9, so it’s not a particularly onerous requirement but I think it’s reasonable to ask for that and then once that’s been verified by credible data provision then it’s just a variety with an added trait…

**Reference 2 - 2.22% Coverage**

PA Off-target effects?

RE It was the repercussions of on-target effects in this particular case. Whole chromosomal deletions and things.

PA That’s not on-target effects, that is what do they call it micro chromogenesis or something, so associated with Cas9 activity in some examples, I think more in mammals than in plants there’s been chromosome breakage events. The fact is if that happens, then you’re going to end up with a variety that underperforms so it needs to be eliminated.

RE And there’s already the facilities, the ways of determining that, that we already have.

PA Yes. All of this (a) is going to be rare, (b) it’s going to lead to underperformance so you’re not going to release any varieties in which this has persisted in the cells, this would not be something I would find troubling.

**<Files\\Koshihikari> - § 13 references coded [14.69% Coverage]**

**Reference 1 - 0.34% Coverage**

Because of the contained use aspect largely. And also, you extract your compound of interest and purify it etc etc, you are not eating the whole plant.

**Reference 2 - 1.10% Coverage**

PA Right, I mean for me it has to be definitely process based.

RE Process based. And why do you think that is?

PA I think because it’s this direct modification of the genome that can give rise to genetic errors. That can then result in unexpected and unpredictable effects.

**Reference 3 - 0.83% Coverage**

Yeah, we did, cause that’s more or less it basically. It’s not about whether it contains foreign genes or not, it’s this direct modification of the genome. Which frankly is imperfect. You know it’s an imperfect science. People don’t really understand all the goings on in the genomes so how can you manipulate it directly. And expect a sort of predictable outcome.

**Reference 4 - 0.20% Coverage**

PA You know so it’s not, it’s not so much as an uncertainty, we know these things happen.

**Reference 5 - 3.04% Coverage**

No, I think well this is the sort of thing that NGO's get themselves you know, is to say no absolutely not it should be a precautionary principle. And then when kind of say well you know, well, actually to sort of you know, to be helpful, what we think is. So, I think for me, if there were to be, if you were. If I was looking at, say if I was, put it another way if I was doing a risk assessment from scratch now. You know what factors would I have in, how different would they be from the existing EFSA guidelines. And the answer is that for me, and especially when we come to genomics there would be much more emphasis on generic irregularities. And for me it’s not enough to say well, you know these are unlikely, it looks alright, therefore it must be alright type of thing. We really must understand what’s going on in that plant at the genomic level and I think that leads the way to all your proteomics, transcriptomics, you know all this sort of products of the genome. To show that they’re the same. You know things have progressed in that field, you know analysing all of these products and I think that would lend itself to a sort of a robust assessment of GM crops. In a way that sort of, the way it has been is just sort of you know say oh well we don’t think it’s going to be an effect it’s like, well how do you know.

**Reference 6 - 0.22% Coverage**

And again, it goes down to this one gene, because the genome is much more complex than we think.

**Reference 7 - 1.24% Coverage**

PA As researchers I think understanding all the genetic errors. I think that’s got to be first and foremost. I mean it seems to me every week that a paper comes out about of target effect or what they call on target effects.

RE Yeah, yeah.

PA You know the fact that damage is done as that sort of healing, and they have a DNA cut and that healing processes is done. So, you know that, that sort of thing needs to be understood and needs to be controlled. I think before you can really start to even think about any commercial applications.

**Reference 8 - 1.68% Coverage**

Are the so-called errors in conventional breeding, are they the same, are they the same type, are they the same origin? Do they have the same effect? You know, nobody knows. For example, there’s this thing about, the one you’re doing say, gene editing, you can access any part of the genome. Whereas in fact you can’t with conventional breeding, some areas are, it’s all to do with the way the DNA is coiled, so some areas are accessible, some areas aren’t accessible. So, in some ways that’s limited then, those, any sort of changes is limited. Now does a change that’s within that inaccessible area to conventional breeding, if that’s accidentally changed by gene editing, is that an error that wouldn’t occur in conventional breeding.

**Reference 9 - 1.69% Coverage**

Who knows? So, I think before we say, oh there’s more errors in conventional breeding, we need to pick unpick exactly what is going on there. So, you know there’s a heap more research to be done there actually. And it, to be honest, really in terms of understanding, how genomes work, how they are regulated, how they switch on, how they switch off, well there’s the who epigenome. you know is a fantastically great mystery. So, there’s a huge amount of understanding, to be honest when I was at [organisation] and people used to go on about exactly what you said, oh well you get more errors in conventional breeding, I was like look, just come back to me in 50 years when you know what you are doing. And I would still say the same thing.

**Reference 10 - 0.69% Coverage**

PA No, for, well no absolutely not, I was being flippant, say you are at a meeting and somebodies trying to have a discussion with you.

RE Ah right I see.

PA You know, the scientists like, oh but don’t you agree, and I would just say no I didn’t. Come back to me when you know what you are doing.

**Reference 11 - 1.22% Coverage**

PA Again, it’s a tweak of one gene.

RE Right.

PA So, they introduced six plasmids in there, so say if you look at Roundup Ready Soya or BT corn that’s one plasma, that’s one GM insert, they used six. Every Time you put in a GM insert, there’s rearrangements and deletions of the host DNA. So, you may have only tweaked on gene, but you have inserted 6 inserts and supposedly got rid them of them. So, hang on a minute, is there any affects from that? You know gone are the days where you say, well it still looks like a Soya bean.

**Reference 12 - 1.85% Coverage**

No exactly and I think there’s, realistically if say the guys at Photoboost want to really sort of like, have social acceptance, this really has to be this these omics technique, looking at the product of the genes. It’s only that sort that of thing that is going to help at the end of the day. You know and it’s, it’s interesting with this, you know, government announcement, what they said is that is things that, the phrasing has to be quite careful here actually. Whether it’s that they don’t insert additional genes or whether they don’t contain additional genes. This sort of so-called foreign genes. Yeah, so even the government says, "GMO regulations will continue to apply where gene editing introduces DNA from other species into an organism". Well, I’m sorry but 99% of gene editing does that anyway.

**Reference 13 - 0.60% Coverage**

PA Yeah, and also, what we are saying is, you’re using all these first-generation techniques, to do your gene editing. So, it’s currently saying, oh we have got rid of all the problems with our first generation by using, because actually you are still using them.

**<Files\\Matla> - § 3 references coded [8.23% Coverage]**

**Reference 1 - 3.81% Coverage**

I’m pretty sure you are aware of this Directive 2001/18, which is pretty old, we all have some issues to deal with mutagenesis- *in vitro* mutagenesis especially in [country], maybe you are aware of that? We are still debating if *in vitro* mutagenesis leads to a GMO under the scope of that [unclear], we are quite far to know how NGT should be regulated you know, 2 years ago, the ECG realised that basically the answer of the ECG was to say if it is after 2001, we don’t know so, it is under the scope but you also know that thanks to [name] and NGT, we have now something of a tool that is now able to produce or to leads to a mutation, single mutation that is equal to a mutation that you can obtain through *in vivo* mutagenesis or just by looking at your crops into the field. So, if you consider that this mutation that could happen naturally are the same than the one you can obtain using NGG then you hope that the breeders who do not have any barriers or any problems to use this technique because at the end you only get this mutation [unclear] by luck or by using mutagenesis, you know that *in vivo* mutagenesis produce a lot- thousands of mutations into your genome so you need to backcross, so with NGT again you can really easily see the hope breeders can have is that just faster everything, all the selection of the breeding process.

**Reference 2 - 3.57% Coverage**

… we should not have a very heavy evaluation process if the mutation is a single mutation that you can obtain with natural process or *in vivo* mutagenesis. If you see the scenario of the new regulation, it could be a new directive or it could also be a regulation that you know that Member States would transfer that to national law the way they want. You know the difference between a directive and regulation? Regulation you don’t have the transfer process, the translation process into national law whilst with directive you have to, OK. I think you’ve got the difference. So it is quite a big difference in fact because in [country] for instance the legislation for GMO is very heavy, maybe heavier than Europe, especially with in vivo mutagenesis case, because basically all the rest of Europe decided that there is no reason to distinguish between in vivo and in vitro while in [country] we are currently discussing a lot and if there is a difference between in vivo and in vitro – the highest [country] court are still thinking about it but realise that maybe they did a mistake because two years ago they decided that there is a difference between in vivo and in vitro if you are using *in vivo* it is ok but in vitro it is GMO.

**Reference 3 - 0.84% Coverage**

As an ex-scientist, a single point mutation, I don’t see the issue of using that. It depends if the gene is from a singular – I don’t know, I mean it depends on the kind of mutation, but a second mutation should not lead to heavy evolution process otherwise you just kill the opportunity to use it.

**<Files\\Njavara> - § 3 references coded [3.64% Coverage]**

**Reference 1 - 0.80% Coverage**

So, that’s their big discussion that’s taking place now. Other than, what’s always been discussed is the very special case of a point mutation that could also occur in nature. But I think in-, what’s mainly been done are other things, yes? So, this is a very unlikely experiment. Instead of this-, our definition would apply. So, to that end.

**Reference 2 - 0.52% Coverage**

RE Well, you probably know that that is the line that people will use when they talk about, certainly plant-breeding technologies. They often draw the comparison to more natural processes.

PA I know. I don’t agree.

**Reference 3 - 2.32% Coverage**

So, this has been a strong argument used by the proponents of genome editing, that you cannot distinguish it from other plants. That it’s not detectable and I think it’s-, this is the ongoing work that we try to change the discussion there a little bit. And now I can see that it’s only being discussed how to detect these plants, because it’s a requirement when you authorise such a crop in the EU to present a detection method. And it’s true that it’s difficult to detect this technique in general and the technique itself it has been used, of course. But that-, this has always been the case for GE, yeah? You always have to know what has been done.

**<Files\\Ofada> - § 2 references coded [8.31% Coverage]**

**Reference 1 - 5.60% Coverage**

Yes, I mean so actually it’s a process trigger but a product assessment. So you would use a certain process targeted mutagenesis or whatever and then at least from our perspective we would say well then you present your product to the regulator and the regulator will have a look and will check what kind of genetic change did you introduce. Maybe you will also need to provide information about the phenotype and about, that you discarded any foreign genetic material which might have been used during the process and then you would get, let’s say, the decision or the confirmation this is a conventional product or this is a GMO depending on what you have been provided. But that’s how also other countries do it, so for example, in Argentina you go to the competent authority for biotechnology, you present either your R&D concept or already a final product and then you describe what you’ve done and then they say, well, you are either conventional or you are GMO and then you follow the respective foods for convention or GM products. So it’s a kind of, how can you say it, a confirmation process or notification process towards an authority so that it’s not kind of the developer who is deciding on the regulatory status but it’s really confirmed by an authority which of course provides more trust I’d say into the regulatory system so in the end, some authority will have a look on the respective products and decide.

**Reference 2 - 2.70% Coverage**

So for us it would be like absence of transgenics, so no novel combination of genetic material as it is called in the Cartagena protocol for example, and then only material from sexually-compatible plant species which would refer to cisgenesis for example and if the result is comparable to what can be achieved by mutagenesis, so that would be like say our criteria and if you provide respective evidence meaning sequence information, for example, towards a regulatory authority and maybe you also describe the phenotype so which, I mean if you work within the plant’s genome, the phenotype should also be comparable to what can be achieved by conventional breeding or natural processes.

**<Files\\Patna> - § 3 references coded [2.36% Coverage]**

**Reference 1 - 0.84% Coverage**

And, I think, do you know, a lot of cynicism kicks in with, this is what you’re saying because you know, they have also discovered that they can’t do very much without being a transgene in.

RE Right, right.

PA You know the burnt toast wheat trial? That’s transgenic. They have used genome editing techniques within it but they’ve also used transgenes and they’re saying, this bizarre positioning that they’re planning to breed out the transgene so it won’t be GM by the end.

**Reference 2 - 0.87% Coverage**

It’s like well, it’s just, like it’s not about what you’re left with. It’s not that like some genes are bad genes and we don’t want to eat food with the bad genes in. I mean maybe there might be some people for spiritual reasons, you know faith reasons who don’t want to eat something that had an animal gene in it or whatever but that’s not broadly what people are concerned about. It’s all the stuff you did along the way, and I think there’s, like it’s hard to talk to the public about science.

**Reference 3 - 0.64% Coverage**

RE Yeah but something that we’ve found, and I think it’s backed up by other surveys I’ve read, by like by the Royal Society is that the application changes attitudes quite a lot. So if it’s for pharmaceutical use, that was one of the projects I was involved in previously, growing, you know, a modified version of tobacco in laboratories.

PA Yeah in contained use.

**<Files\\Salari> - § 1 reference coded [6.14% Coverage]**

**Reference 1 - 6.14% Coverage**

Yes, clearly there's a technical difference between the techniques, the way they work. The idea is that if you could, from a theoretical perspective, say okay, let's change one base pair and the whole genome of the plant, and this, I don't know, knocks out a certain gene. Yes, on an intellectual level, it is a different situation to a certain degree, because it is then used as a comparison to, you know, this could happen in nature. You could have a mutation also in nature, but yes, it definitely plays into why some of the actors are rethinking it. Because it seems a bit of a less drastic intervention. Clearly it remains genetic engineering, it remains a technical intervention, but less of a drastic one, but what is also to be considered is that in many cases it will not stay with this one modification, with this one change of base pairs. If you look at plant genes, you have many copies of different genes, and if you would want to achieve a certain trait, in many cases, you would probably knock out all of the 10 copies, for example of a gene. So you could argue that this is already quite an intensive technical intervention, and this justifies the same level of precaution and the same level of regulation that we do have currently, and what we also say is that it is important to have this risk assessment. But in this risk assessment, you can have a different risk assessment approach, depending on the degree of the intervention you have. But it would be dangerous to just assume, because it is a technique that you can use to only change one base pair, it is always used in this way and therefore justifies to skip all of the risk assessment procedures.

**<Files\\Sharbati> - § 5 references coded [14.79% Coverage]**

**Reference 1 - 1.85% Coverage**

RE Excellent. There you go. What do you think are the biggest challenges for the European food and agricultural sector at the moment? And, maybe, particularly around plant breeding?

PA Ah, yeah. This mingle between new breeding technologies, metagenesis and classical breeding, to draw a line here, I think. I think it’s-, they have a hard time in drawing the line. Currently, they see all new genomic techniques as GMOs, as you might know.

**Reference 2 - 3.06% Coverage**

But you can also do it completely without integrating the tools into the genome in the first place. So, what we are doing at the moment is that we are just using the isolated Cas9 enzyme in a combination with the guide RNAs, the so-called r-genecomplex, the RNA-guided nuclease complex, and bring them in as a protein. For example, in protoplasts or in somatic embryos-,

RE Right.

PA And they cut and they are dissolved by the cells in 72 hours and they are gone, so you don’t have any trace of editing and have just imitation. I have never used recombinant DNA because it’s just a protein and an RA which has then gone and you just stay with the mutation. And there is no chance of integrating any foreign DNA into the plant.

**Reference 3 - 2.74% Coverage**

Well, in Europe, I think, now with genome-editing tools in hand we can really make cisgenesis as it should be. So, introducing a gene that is crossable at the direct place where there’s inter-crossable species. And, as soon as we have done this, and there’s no CRISPR or any other genome-editing tool left in the plant, I think it should be considered as natural and not as a GMO. And this is where the law-, the line should be drawn. So, cisgenesis in the [unclear] should not be regulated as a GMO. And if I have my crossable gene integrated somewhere else in the genome, for example, with backbone, this is still a GMO because I have foreign DNA in it.

**Reference 4 - 1.99% Coverage**

PA The backbone DNA. For example, the right or left border of the T-DNA from agrobacteria. So, if I have really no foreign DNA in my plant, then from my point of view, it’s a natural event and not a transgenic or a GMO.

RE Okay, so you’re still keen on this distinction. You think this distinction is still important and should be the kind of, the bedrock, if you like, of the regulatory framework around these things?

PA Yes. Yeah. From my personal point of view, yes.

**Reference 5 - 5.14% Coverage**

PA That we never know about potential off-target or additional on-target effects. Whenever we do breeding, as a good example, we can use irradiation breeding or using chemicals where we have multiple off-target effects. Hundreds or thousands more than we have seen with CRISPR. Which are completely undirected in the com-, and then they distribute over the whole genome. And also, when we are doing breeding we have retrogression. We have realignment of chromosome parts. We have introgression of crossable species into our species as well as-, so, variation is taking place whenever we do breeding. And, with CRISPR, it’s even more predictable what might-,

RE Right.

PA Happen. And, in most cases, you never go with the first direct mutant into the market. So, you have at least some refinements that’s in there. With backcrossing or crossing, where you lose most of the off-targets, if that happened at all with CRISPR. And with classical breeding, or classical mutagenesis, you have to stoop but you never get rid of all off-targets, so you stay, your classical breeding, you stay with off-targets and-, which you just accept. With CRISPR, you have a good chance to get rid of all of the off-targets. If it ever happened.

**Name:** Challenges

<Files\\Chandra> - § 4 references coded [2.72% Coverage]

Reference 1 - 0.38% Coverage

So stepping back from all the short-term issues that we're currently facing, the, you know, the one that just like happened in the last year and in terms of, particularly the repercussions of the war against Ukraine.

Reference 2 - 0.69% Coverage

But in terms of like, you know, basically like maybe before, before this year my answer was maybe different and that would be that there is kind of twin challenges when it comes to agriculture. One of them is the need to, I mean the corny way of putting it like I say it's corny, maybe it's my ecological scientific background is we need to shift towards working with nature not against it.

Reference 3 - 0.26% Coverage

That's the kind of the line that kind of summarises the greater challenge for those who perhaps don't have an understanding of ecology and so on.

Reference 4 - 1.39% Coverage

We do, if we try and solve the problems in agriculture without addressing what, how we need to, what we need, what we're producing and what we're actually doing with it. So the waste side, the obesity side of things, the malnutrition side of things, we, and also obviously the emission side, you just go down the wrong path. So it's very much those like dual challenges yeah, and we see at [organisation] that a transition to agroecology is possible, alongside dietary changes as a solution, though we tend to position it more as a kind of, we look at it more in terms of modelling and so on as a kind of a scenario but we're not necessarily saying it's just, for example, the solution we wouldn't say would be everyone needs to adopt organic standards. We wouldn't go so far as that.

<Files\\Ginpu> - § 1 reference coded [7.02% Coverage]

Reference 1 - 7.02% Coverage

Yes, lots of challenges, lots of uncertainty. At the moment, I would say the biggest challenge is risk and uncertainty; impact on global markets and supply chains, geopolitical, all these things that we wouldn’t even have thought about a few years ago have now suddenly come into view, so risk is a big one. There's obviously the climate and the nature challenge as well, which are political priorities, but they're also very important to the marketplace and the financial institutions that support agriculture as well. In summary, I would say in the short to medium, probably short to long term, it's probably uncertainty and risk is a big one as the climate is changing and there are more pest and disease challenges, more agronomic challenges coming in as well as the market. But then also meeting the net zero aspirations of the market and government will be an increasing challenge and also the nature as well. So restoring nature is going to be a big challenge as well in terms of cropping.

<Files\\Jata> - § 1 reference coded [3.36% Coverage]

Reference 1 - 3.36% Coverage

Well I think we have the challenges of the climate change which we actually face right now, me being almost baked in my apartment right now. So, from the other hand, I think that Europeans, they are not very much concerned about the climate change, not as much as in other parts of the world because we still have the access to all the food products in the stores and we can still choose and buy, the price is going higher but still it’s available so, I think for the European consumers, what actually was I think visible for them was the pandemic and the current geopolitical situation, I think these two are for the Europeans right now more visible from the perspective of the food security than the climate change.

<Files\\Njavara> - § 2 references coded [3.73% Coverage]

Reference 1 - 1.89% Coverage

So, we have to do big changes in agriculture. When it comes to mitigation, then it’s more or less focusing on smallness and larger areas. So-, and of costs. It’s what we always hear. So, when we talk about agriculture and if we talk about our idea of agriculture that’s more organic, more close to nature, less focused on pesticides, less inputs and things like that, then we always come to the discussion to feed the world. You know? So, that’s-, I think this has been a wrong path in the past in Europe. That we need to feed the world. And, in the end, I’m really struggling with the issue that we could feed the world already, yes? So, there are more people suffering from hunger each and every year, these last years. And, yeah, so-, in Europe, it’s climate change, it’s nitrates in the groundwater.

Reference 2 - 1.84% Coverage

Other emissions, like ammonia emissions, fine dust and, of course, it’s also livestock. Livestock is very essential for us. And we have to reduce our livestock populations. We have to change consumption patterns. We have to work on food waste. So, it’s really-, food culture is very complex in the end, also. It’s not as-, not like. So, we need a complete change of the agricultural model. Of the world food system in the end. But I think it’s even more challenging than just the energy sector is. Yes? So, we have a clear concept for the energy sector in [country] but that’s easy when you calculate it. So, it’s just switching off some nuclear power plants and building some renewables instead. But in agriculture, it’s very interlinked with so many things and it’s so international…

<Files\\Ondel> - § 1 reference coded [0.61% Coverage]

Reference 1 - 0.61% Coverage

There are many challenges. For example, like I’ve been to conference in [country] as I mentioned and many people are concerned about acrylamide.

<Files\\Patna> - § 1 reference coded [0.84% Coverage]

Reference 1 - 0.84% Coverage

I mean obviously climate change, you know, the climate emergency is a challenge, you know, absolutely for everybody. But I would say specific to agriculture, it’s probably soil. The state of the soil is, is a terrifying emergency just you know, not long away and it’s not getting the attention that it should. But actually, if you’re looking at kind of, what’s wrong with the system, I always come back to waste. We waste, I think the estimate is about a third of the world’s food.

<Files\\Salari> - § 2 references coded [4.49% Coverage]

Reference 1 - 1.73% Coverage

Well, in general, one of the major topics would narrow it down to soils and inputs. The one big problem we're dealing with is the soils. You might have heard of this UN report that says there are only 50 harvests left and this relates to soil degradation. This soil degradation is caused by an over input of nutrients into the soil, overuse of pesticides that leads to soil degradation. Soil degradation leads to less fertile soils, which then would decrease productivity.

Reference 2 - 2.75% Coverage

So for organic farming it’s also an important point to maintain the soil health, to not go for the short term maximisation of yield, but rather to look at long term perspective to also ensure the long term food security. Food security is also a big topic, but [farming] movement sees it a bit more in a long term perspective. And then, of course, in relation to input is also the overuse of pesticides, which leads to a degradation of biodiversity, of insect populations and so on which are connected to farming. Some farming systems will see the farm more as a factory where you want to have as controlled an environment as possible. Whereas for organic farming, it sees the farming more as a part of the environment as well and the living environment.

**Name:** Considerations for regulation

**<Files\\Ambemore> - § 9 references coded [21.76% Coverage]**

**Reference 1 - 2.05% Coverage**

I have been involved with the debate of new breeding techniques and in [year] [organisation] [activity] because we wanted to push on the Commission to achieve that the Commission move on a decision to clarify the legal statute of the new genomic technique, new breeding technique because, at that time, the expert group of the Member States had, already in 2009 or 2011 produced a broad report and out of this report the sector would have expected that the Commission move and did some clarification but, the Commission did not move, but, since [year] we have pushed regularly on the Commission to move.

**Reference 2 - 1.75% Coverage**

On the procedure, what we have decided, we have a clear position, we want to resolve the issue of the mutagenesis technique, when they deliver a product or seed or a variety that is comparable to a conventional seed and the way we want to do it, we want to revise the GMO Directive, but to have a technical amendment maybe inside the definition but mainly in the Annex 1B where they made this derogation for this old metagenesis technique, we want to amend with the new metagenesis technique because, the derogation is already there.

**Reference 3 - 0.91% Coverage**

We have asked since 2016 to make this clarification as first step as a prime example. We notice that in the Commission Report, the Commission has opened the debate to cisgenesis and we know that in cisgenesis it’s maybe much more GMO because you use an external DNA…

**Reference 4 - 7.58% Coverage**

Also, we have another concern because even the regulatory frameworks are not the same they at the level of the farmers and industry connected, it is the issue of the patent because, in the patent issue, [organisation] have always patent plant and animals and we have always opposed to every system of intellectual property based on patent. We want to keep the system of CPVO and mainly we want to keep the gene pool open to every breeder and every farmer because our Members follow the line that our system has been the best in the world because in the EU we have a big turnover in the catalogue and we have to keep breeding also on minor crops such as fruit and vegetables and other minor crops and our Members take the view that if they go for a patent system we will be like in the United States… it will first have an impact on the number of breeders because, the small breeders or the family breeders will not be able to pay all the lawyers to manager the registration in the patent system. This money will be used for lawyers and not invested in research for new varieties. They believe that will reduce the number of breeders and it will reduce the competition in the sector and it will reduce the number of varieties put on the market and at the end it will not be beneficial for the farmers and for the consumers and also, because the scope of the patents will go beyond the seed, it will go up to the consumers because, the patent holders it can take also a fee on the beers that contain the hops that have been genetically modified and covered by the scope of the patent. We have always hesitated and when it comes to the debate of the Commission on mutagenesis and seed genesis for example the line is that we don’t want to talk about seed genesis as long as we don’t have resolve the scope of the patent for the crop obtained by cisgenesis. Then I know well that [organisation] will come with the system of platform for the licensing and things like this but, well I have to tell you blankly that the farmers they don’t want to go to this kind of negotiation they reject patent on plant and animals and they have a strong line but they are aware that today patent are granted but they don’t – they see risk for themselves and they see risk for the food system and for the consumer at the end.

**Reference 5 - 3.85% Coverage**

Yes, because in the CPVO this is also a system of intellectual property. You put a new variety on the market, you have to prove that the variety is distinguishable, homogenous and stable compared to an existing variety [indicator] you compare it to [an indicator], existing varieities and after that you have also to elaborate and to demonstrate that the varieties has additional value for agriculture and for technological use and when the variety has passed the system, it can be registered to the National and European Catalogue and benefit from the single new market. When it is protected, the breeder receives also royalties. I would say that the level of the royalties it is less money compared to the patent system but, every breeder, every farmer can use this free without negotiating anything with the breeders to create another one. That is plainly according to us a system that supports a lot of innovation, and it is a lot of innovation is proven by the number of varieties coming in, in the catalogue and by the EU global position in the seed sector worldwide. It is why in [organisation], they want to keep it.

**Reference 6 - 0.83% Coverage**

… for us, it is to go in the scope of the current system. We do not need to create another scope and again maybe we need to create another scope for metagenesis technique the same, we don’t need to create another regulation with the kind of risk assessment.

**Reference 7 - 2.92% Coverage**

What we have agreed to do is to increase the transparency. For example, in the European Catalogue, when it is a hybrid variety you have, if I know enough about the system, you have indicated the parents of the hybrid. We believe that in the same way the result disclosing the information of the breeder to another breeder, the information, the process of the breeder, that we should find a way to give in the Catalogue, information, for example, if it is a new mutagenesis technique, in case of in the supply chain some supermarkets do not want that the farmers use this kind of variety – they can know that they cannot use it to satisfy the demand later in the chain. This information should maybe very general because of course we want also to protect the recipes of the breeders that we believe it should be amended in the way we put information in the Catalogue to provide information.

**Reference 8 - 0.91% Coverage**

… we are not asking to label it on the package of the seed but just that the farmers have to know in case his buyer does not accept it, that why to label something that you cannot protect in a seed or in varieties for us it makes no sense. It is disinformation of the consumers.

**Reference 9 - 0.97% Coverage**

… because if they would have proceeded only by explaining that today maybe 70% of varieties have been obtained with new mutagenesis techniques, because the techniques have evolved so much since 2001 and have become legal and maybe technique amendment to the GMO, we would have loved this way to do…

**<Files\\Chandra> - § 20 references coded [20.21% Coverage]**

**Reference 1 - 1.25% Coverage**

But particularly I guess the way that I tend to see things is that you have like almost a pathway that the technology takes society down, and too often we're not really thinking about what that pathway is and where it's taking us. It's just like, it's the, so much focus is in there now. Yeah, like genetic modification is like, is a good example where I'd love to see more research that was looking at what the, for example, the intellectual property rights side of it. What that actually will, what the repercussions of that are. It just like has huge implications for that pathway. But there's not been much research into that and as a result you get a lot of conjecture which isn't necessarily helpful.

**Reference 2 - 0.74% Coverage**

But in a hypothetical world where you know you could pick and choose exactly what the, what regulation would look like and you didn't have those options, then like me, it sounds like based on my limited knowledge that you could have assessed the original regulatory framework that was drawn up was a long time ago and could be updated, and could be much better, much more, you know, in terms of there's kind of two areas.

**Reference 3 - 0.54% Coverage**

And legally I'm worried that's meaningless based on the evidence given by a lawyer. It’s the only evidence I've seen though presented by someone in terms of legally what does that mean. Effectively, there's no timescale to it. So what in, you could argue what in, like 10 million years it’s possible to…

**Reference 4 - 1.25% Coverage**

What that actually looks like in practice though, beyond what I’ve just said, I'm not entirely sure, but I guess what I'm particularly interested in is how do you have a… at the moment the proposal is to have a two stage approach, so that effectively, particularly novel changes get an extra level of scrutiny in terms of regulation. But a stepped approach sounds reasonable to me. I'm just worried that the current approach is basically being rushed politically, is not evidence based and hasn't really been thought through very well. And I'm really hoping the EU takes a much more evidence based, careful approach. It really does feel so rushed. So, so, so rushed what the government is trying to propose.

**Reference 5 - 0.37% Coverage**

The area that I'm really interested in is the other risks, the kind of like the logical risks that are hard to know they exist around issues like how is the technology going to be used? Who owns the technology?

**Reference 6 - 2.97% Coverage**

No, so we spoke to someone from the regulatory policy committee and effectively it's considered incredibly bad practice to not be evidence based in drafting legislation and therefore it's fairly unprecedented, I believe, to proceed with a bill without sorting out the underlying evidence underpinning it, particularly on, you know, that economic impact side. And yeah, it's just another indicator of how political this is because, yeah, there's huge impacts to businesses that could easily be solved. You can still have the purpose of the bill which is you know, to loosen regulation around gene editing and reduce that economic impact. But currently the fact that it's not looked at will cause a huge amount of risk to businesses, completely unnecessarily, which is a whole another area which is just mad. That’s around traceability and supply chains and auditing and the fact that there's too many markets that are reliant on separation and will continue to be for years even if things change and the government completely ignored that, and given the ability to have supply chain auditing now, you know, so advanced and entirely possible and not expensive and it's not like, yeah, it's just mad that they didn't look at it, and as a result, it's not being legislated as a mandatory. So yeah, so that's, sorry I'm going off on a tangent because there's so many sides to this. The business side of it is just a whole area that's just completely mad that the government haven't looked at in detail. And as I say, yeah. So you would think that they should cause the bill and check all of the economic impact side of this and then amend the bill slightly and proceed. But for some reason.

**Reference 7 - 0.62% Coverage**

Well our concern is that it could, and there's not, there's no, with our government is not concerned about it, there has be no research into it. There's been no debate about it, they're not brought in experts who can say what will happen. It’s just, it's like a vacuum of, we just hope it will be all okay and that it will all be in the public good.

**Reference 8 - 0.78% Coverage**

So what we would, it's a little bit challenging because it's, we don't normally have this for technology but the ideal would be that technology that had grave risks would be steered in the direction of the public good if you know, and at the moment for our point of view is that breeding in plants and animals has not, generally, the market has not pushed breeding in the right directions and so why would it suddenly do it with gene editing?

**Reference 9 - 0.83% Coverage**

So I mean, it's really interesting to look at that like in terms of animal breeding which is what the Nuffield bioethics public dialogue did and the public dialogue that's going to be a report on the one this year will be published soon, that was meant to be published this week. So that looked at that, which is you know how, kind of in a way, like what are the, effectively what are the commercial uses of this technology and do they align with what the government want?

**Reference 10 - 1.54% Coverage**

So what I'd like to see is legislation which was more science based when it came to the safety side, and was kind of basically only allowed things through that were for the public good so that there would be like a panel looking at that in terms of the environmental side, but obviously that would be quite a novel thing to do with the technology. So although I think Labour have proposed another amendment along those lines, I don't think it's going to go through. But it's just the fact that the government are ignoring the commercial drive and the commercial drivers towards roundup ready crops, which I believe you can do through gene editing depending on the species, theoretically, and also in animals it's to do with increasing productivity which is a problem for animals because currently most animal breeds that have been pushed to the limits already, and I believe.

**Reference 11 - 1.10% Coverage**

Yeah, I mean in plants for example, one of the concerns that we have is that the government's legislation, the line that's being drawn around what will count as precision bred and what will count as genetic modification does seem a little bit kind of hard to understand as to what that would be in practice legally, legally is what really matters because you know you can obviously, if you're a company and you want to dispute that your product, whether or not it fits the precision bred, it's what you legally argue, it’s what really matters at the end of the day as opposed to what the government hopes for that legislation.

**Reference 12 - 0.20% Coverage**

And then, yeah, that line seems so woolly to me and so lacking in science in terms of what, you know, what does it?

**Reference 13 - 1.29% Coverage**

I've got no idea what it is going to mean in terms of like, particularly for accumulated edits. I feel where the line will be drawn, and as a result it does seem very likely that you're going to get, particularly with accumulated edits, plants that may well be so competitive that they could even end up, this is the worst case scenario, becoming new invasives, you know, entirely possible theoretically I assume, and yet presumably fairly easy to research and look into in terms of you know, looking at literally the ability to out compete wild plants, you know. There must be ways of doing kind of, like I'm not, it's just it's kind of a matter of just being aware of that risk and having kind of some simple ways to look at it.

**Reference 14 - 1.39% Coverage**

You can't manage it entirely. So yeah, so there's that side of it particularly in terms of productivity which is obviously relevant to this. Just being aware basically of that ability to out compete natives, and then particularly in terms of trees I think that's a quite interesting one. And then the other side of it is, life state, persistent crops or any crops that are designed to work with a product, and just looking at history as to what that's achieved, short term can be really great but long term, I kind of see it as two things. One you have like the really obvious kind of clear thing which is like, you rely on it so much that you end up with super weeds and then your whole farming system is based on that one product and as a result you're kind of, as a farming you’re stuck.

**Reference 15 - 0.70% Coverage**

Yeah, and then in terms of, I mean like the differences with other breeds is that you have, you know, which haven't been created through biotechnology, is, and this is again, not an area I understand in detail and I would love to see a kind of neutral analysis of it is that you, my understanding is you are more likely to be able to patent a plant if you've used a novel technology to create it.

**Reference 16 - 2.36% Coverage**

And intellectual property rights is a really important part of agriculture. They're not, you know, bad in of itself but plant patents are generally like, for example, the NFU came out against plant patents a while ago. So yeah, what does it, what’s it going to mean if we have genetic engineered crops patented? What's that going to, you know, going back to that path, that pathway of society. Where is that going to take us? It may stop wasting. We may end up. We start out with some really nice things that come out from publicly funded research institutes perhaps, but maybe anything will be brought up. Patents brought up, you have issues with patented around the actual process itself and who actually owns the, you know, the technology itself, you know for example, like the crisper, yeah, I think they said the legal side of it is so complicated that it's like levels of, I know there's levels of patterns of the actual technique and then there's levels of patterns of the traits. Yeah, and my please is just simply that there is research into that so that we understand the implications in terms of the market and where it will take us long term. I'm not, I wouldn't, I'm worried about it, but I would love it, I’d love the case to be that it's all you know, witness worry. It would be fantastic if you don't know if that happened.

**Reference 17 - 1.42% Coverage**

So there's a whole area of work there which is very much on that economic impact side and trying to make the case that there are some simple changes that could be made that would enable everyone to get what they want, which is, yeah, the government to get deregulated gene editing through, but organic businesses and those who trade in such a way they would need to prove that their products don’t contain gene-edited products would be able to continue to trade. It's all entirely possible, but it's not being addressed. So that's the other area we're looking at, but that's very much from a business angle. So for example, we wrote to the new secretary of state about that asking for a pause in the bill for the missing evidence on the economic impact to be filled in, which may happen, we'll see. Yeah.

**Reference 18 - 0.34% Coverage**

So who will own, you know, what will the difference be from those products in terms of ownership and intellectual property rights compared to if it hadn't been achieved through biotechnology?

**Reference 19 - 0.24% Coverage**

And what are the implications of that, and so the intellectual property right side and then the other side of it is just how much is it?

**Reference 20 - 0.28% Coverage**

Yeah, because it’s not what's happened in, the thing I always look at is in, up till now in breeding what has happened? And why would that trajectory change?

**<Files\\Ginpu> - § 2 references coded [9.51% Coverage]**

**Reference 1 - 4.36% Coverage**

RE How would an ideal regulatory system look from your perspective, for new genomic techniques specifically?

PA Ideally, it would be UK-wide and the regulation would sit within the UK, and then all the devolved nations would take part. It would just be in place for all the devolved nations. I suppose it's a bit like plant breeding where you’ve got these existing measurements; distinct, uniform, stable regulations and things like that. I'm not 100% sure on this, but I guess you would build in at that variety breeding parts. I suppose it would also include how much it goes into the other populations of plants.

**Reference 2 - 5.15% Coverage**

RE I can tell you that the difference between the two big camps that have formed about this issue are generally those that want to see the GMO policies we inherited from the European Union, the 2001 directive kept, and those who want to see gene edited crops if they are reasonably simple, so like a gene knockout. Like SDN1, they would like to see those go through the standard variety registration process, so basically, there's no extra risk attributed to them, you’re going to have to go through the process.

PA We're in the latter camp. If you're not introducing any new genetic material, then you would use existing techniques. Our argument is it is speeding up the breeding process rather than introducing new material.

**<Files\\Illabong> - § 10 references coded [45.39% Coverage]**

**Reference 1 - 3.95% Coverage**

What the European Commission has in mind is that they will move gene edited crops out of the 2018 directive for GMO’s. I don’t know how this will work, honestly I don’t know how they want to do this. Either they are GMO’s or they are not, trying to intermingle there opens up the floor for a lot of controversial issues in this sense to what extent is this an implication for example for labelling, will this still be required and at what level and traceability, so this basically adds at least what I have seen so far another issue of complication and not solving problems but I might also be mistaken because may be they something in their hand that no one else knows. I would not be surprised.

**Reference 2 - 13.75% Coverage**

Ok yes so some people suggest that you move the New Plant Biotechnology into Annex 1B and for making an exception for this you need to get these changes approved by the majority of Member States – I don’t see this as being possible at this point in time. Getting this new Regulation that might be something for the time that people get busy with trying to understand what this may imply but it basically still does not solve the problem you still have to go through the approval process via the comitology procedures so EFSA will give a positive signal and then the Standing Committee if they would be willing to differentiate between GMO’s and Gene Edited Crops, I am not so sure for them it would still be a GMO or something similar and the voting behaviour will not change so having another regulation but not having a change in the decision making procedures, this does not solve the problem. Now the one thing that might be interesting is that if someone else started a new court case so that is a legal possibility. There was a court case that came out of France where the local Courts decided that they needed clarification with regard to GMO’s. Now you could think about a new court case in Germany or The Netherlands or somewhere where a farmer just cultivates let's say Clearfield technology and says no, that’s not a GMO. Then the local Court has to sue him and the local Court says yes, according to the evidence that we have available it is not a GMO because its being based on the evidence being presented to the French Courts now we have the German Court and they may get other evidence and then that Court may say it is not a GMO. Then of course the Environmental Group will pursue that Court decision and then it might go back to the European Court of Justice and then with other evidence being presented they may change their result because the European Court of Justice says it is not a fact finding mission. They can only base and that is what they stress explicitly, they can only base their decision on the evidence presented to the European Court of Justice. They cannot question the evidence the French Court presents. So the German or Dutch Court may come with some other evidence and say no it is not a GMO according to our assessment and they are perfectly safe. Then the European Court of Justice may come up with a different decision. That seems to me the only way that can come out of this conundrum.

**Reference 3 - 4.16% Coverage**

In part yes, If the UK goes ahead and develops your rice variety or wheat variety that they are doing a lot of that, right, getting them approved for cultivation in the UK and then the UK applies for approval for import into the European Union, I think you have a fair chance that this will be granted. No that’s not correct, it will be granted so the food safety authority will say yes, the Standing Committee will say no, the Peer Committee will say nothing and the European Commission can decide and they will say yes because of EFSA then after 4 or 5 years you have your approval for export into the European Union. While you may get it just within 1 year or even within 6 months if you want to export to the US or to Latin America.

**Reference 4 - 5.27% Coverage**

RE So what you’ve discussed there comes on to a question I have a bit later in my little list is about the relative influence of different institutions and organisations in this process of either making or re-affirming regulation around biotechnology in Europe. I would like to share with you my screen because we put together a little kind of map so that we can understand this a bit better and hopefully visually display two people, what this looks like, so if I go to share content, window 6 – can you see, so this is what we have at this point and we’ve discussed with some people already this image and I’d like your take on it and whether or not we’re missing anything big. The European Commission is rather a large entity but EFSA perhaps – would you add EFSA as a separate entity here?

PA Yes – they have a GMO panel on EFSA – they have a lot of say in basically the safety assessments that applicants have to implement.

**Reference 5 - 0.67% Coverage**

PA They also access dossiers this can be fast or less fast and this is basically in the hand of EFSA.

**Reference 6 - 1.54% Coverage**

RE Right, I see. Do you think that the picture changes with respect to gene editing versus what I will call first generation GMO. No you think not.

PA It has the same issues, the same debates, the same groups in favour and against, nothing has changed in that regards.

**Reference 7 - 9.33% Coverage**

Yes I would completely scrap 2001 – it only came into place when they started EFSA in response to the Mad Cow Disease. So before food was a normal food before EFSA came into place and the whole new procedure of assessments. That was minimal harmonisation, basically Member States decide on their own about the safety of the food etc., and then if it was approved in Germany you could sell it all over Europe and if it was approved in the UK you could sell it all over Europe. Then came Mad Cow Disease and there were different standards. In mainland Europe you had to increase the temperature I think from 90-95 or a little bit more and then this destroys the prions that cause Mad Cows Disease. In the UK the requirement was only to heat to 75 degrees which did not destroy the prions. That’s why it ended up in the feed for cows and you get the Mad Cow Disease. So they say based on this experiences they said we don’t want to have this being regulated at individual Member States, we want to have something that is harmonised across Europe. They did establish EFSA. European Food Standard Authority to identify and harmonise standards. That was mainly driven by the Mad Cow Disease. At the same point in time the debates on GMO’s were coming up when the Mad Cow Disease was there and we had the soft drinks scandal in Belgium and in France with dioxide in the coca cola bottle. You also had the HIV Aids tainted blood scandal in France. There were many things that came together at the same point in time. EFSA and GMO’s came in and the Member States said okay, we want safety regulations for GMO’s as well. You’re talking about novel food regulation.

**Reference 8 - 1.31% Coverage**

Yes – the one that has been approved is the Monet 10 the maize event for maize cultivation in Spain and a few other EU countries. This was approved under novel food and was not approved under the reformed process not another 2001-18.

**Reference 9 - 4.01% Coverage**

RA I didn’t realise that, that’s quite interesting that it went that route… did gene-edited crops change anything in terms of co-existence policies or is that more in terms of GMO specific if can we separate those two terms for the moment.

PE It’s all political and nothing to do with science so as long as there is another GMO policy then most countries will also apply the co-existence regulations. So there are minimal harmonisation policies so Member States are free to implement co-existence policies.

**Reference 10 - 1.39% Coverage**

Even for Monet 10 – you don’t. They say ok we have legal and we have laws for trespassing for damage on neighbouring land etc., all of these issues are already covered by standard law so you do not need any additional laws for damage to property.

**<Files\\Jata> - § 4 references coded [11.07% Coverage]**

**Reference 1 - 4.22% Coverage**

So, from the perspective of the scientists, I think what the UK has done is a very good move, I think for scientists the most important are field trials as the results obtained in the laboratory are not obviously the same as in the field so, this is the first thing that the scientists I think would like to have it more easy so, I think the administration costs and the time and the whole procedure of admissions of such a field trial would be beneficial to shorten it and loosen it up as it is right now in the UK.

RE Yes, yeah because we don’t, in the UK we don’t know what the route to market looks like yet and that kind of stuff, but field trials have been made easier so, you see that as being a logical first step?

PA As a stakeholder who is you know, talking from the scientist perspective, I think that’s the most important part for us and actually the first one in all the process.

**Reference 2 - 3.57% Coverage**

Okay well, I’m not sure because the Commission has all these surveys, and they open different consultations on different topics, and they all go through the same kind of timeline or procedure, and this is how they work. I don’t think you can change very easily the way the Commission works but we use the UK as an example of what can you do and how much faster can you go with the regulation if you did not have all this restrictive, well not regulations but these authorities above you and you have to go with their administrative procedures to contribute to their initiative which was actually asked by the Member States and the Member States asked because of other stakeholders and so forth, so it’s all, it’s not as one country, it’s all the Member States.

**Reference 3 - 0.87% Coverage**

You know, this opt out option, even if the regulation would be in place, that let’s say the scientists would be happy with, still each of the Member States can regulate it differently.

**Reference 4 - 2.41% Coverage**

I think they should be looking for more scientific information than the emotions and beliefs which are also very important but not important for the regulation. Nobody asks of the beliefs of people while building, I don’t know, a space ship or something which requires technology, no one asks you as a consumer what do you think of that I mean, or what is your belief, will it be closer to God or not, I mean these are the other kinds of questions I think, that shouldn’t be involved into the regulatory process.

**<Files\\Kebo> - § 7 references coded [15.64% Coverage]**

**Reference 1 - 0.71% Coverage**

… but this is part of the problem within the EU at least in particular a lot of the compounds that people have been applying are being declared too unsafe to use but genetic substitutes are not permitted so it is a oneself in the foot-shooting experience.

**Reference 2 - 3.50% Coverage**

Now I think it’s reasonable to ask me to verify that where the DNA went in that we put in did the job or from elevating these… or improving these traits. Is there any plausible way whereby that could have changed regulation of adjacent genes in a deleterious way, we’d have to handle that with written arguments. When one makes transgenic plants there’s a phenomenon called somaclonal variation and so this can give you changes in performance, so usually they’re deleterious so you have to do field trials to check that there’s no effect on yield…

[redacted reference]

[redacted reference]

[redacted reference]

**Reference 7 - 1.79% Coverage**

PA And I come back to this community outrage thing. The key thing is that anything like that is made available in a manner by which people can say thanks but no thanks.

RE So you would be a fan of labelling?

PA Yes but I’m sure all the lab. grown meat and whatever is going to be labelled as having come through, being produced differently. People are concerned quite rightly about welfare of farm animals and animals grown for slaughter and so any product that removes that is going to have a pretty decent sized market I think. Of course you’ll have the…even better, we hardly eat any meat these days because I don’t like the impact of it on the planet.

**<Files\\Koshihikari> - § 10 references coded [12.48% Coverage]**

**Reference 1 - 1.35% Coverage**

But there’s kind of a trap there in that, because it’s science-based risk assessment in the EU. Then both government and NGOs are arguing on a science basis. And this used to drive EFSA crazy. Right there would be some paper out about BT and ladybirds and Poland would say, oh look here’s some new evidence, we are not growing GM crops. And actually, it was sort of a societal decision that they had made. Rather than based in the science. So, in a way you kind of get trapped into that science and it was only recently with the opt out clauses that came out. I don’t know if you know those.

**Reference 2 - 0.32% Coverage**

It was yeah, erm, that erm, that countries could say, well look we just don’t want GM crops, so you were always trying to shoehorn science.

**Reference 3 - 0.35% Coverage**

And there’s a real trap with the GMO's actually that I think both NGO's and everyone that’s fallen into, is purely looking at it through a science lens.

**Reference 4 - 3.36% Coverage**

RE So do you think there is a route for approval of let’s say, a gene edited crop, can it ever satisfy enough criteria to be, to be made, well for the product to be in the market basically.

PA As an agricultural product, no. And I think there’s two things in there really, is, one is the deliberate environmental release. And then also the health aspect of consuming say the whole plant. Now I think that you could, for example, there’s one, like the fish oil in Camelina, do you know that one? It’s a UK thing, so you know, I have said, I said this years ago why don’t you just grow it in a greenhouse and extract the fish oil.

RE What did they say?

PA It wouldn’t be economic.

RE Wouldn’t be economic because of the size, do they need a field scale, ok.

PA So, you know that’s the sort of thing and I think it’s really, and I mean I would call that fish oil Camelina a pharmaceutical crop. Because it’s to produce a bioactive compound. But there’s no reason why you couldn’t do that in my book. I mean, hilarious originally, they were doing it in oilseed rape. DEFRA just said we are never going to let you have a field release of this. Because it would just so you know, contaminate everything.

RE Well yeah there’s grain all over the country.

PA Yeah, and you see oilseed rape, not so much down here, but in Essex and stuff it grows at the side of the road, the middle of the road. You know so no, it’s not going to be contained in any, any respect.

**Reference 5 - 0.47% Coverage**

If I was looking at, say if I was, put it another way if I was doing a risk assessment from scratch now. You know what factors would I have in, how different would they be from the existing EFSA guidelines.

**Reference 6 - 0.84% Coverage**

That doesn’t get you out of the environmental deliberate release though. And I think that is altogether a different problem, because it’s very hard to see any sort of coexistence, it may work, and I think it does work that well in the US, where they have got huge fields. But here in the UK we don’t have huge fields and of course you know, we have the organic sector.

**Reference 7 - 2.01% Coverage**

PA So, coexistence is not possible and also that potential for contamination means that if something does go wrong there’s no, there’s no recall. Once it’s sort of out there it’s out there. I mean the one I always take about is herbicide tolerant grass in the US. And it was basically it was, at a field station and they were drying the grass seed and a gust of wind came, blew the seed and now they’ve got herbicide tolerant grass growing everywhere in the US. And there’s no way they can get that back.

RE How did that, did they breed the grass or did it cross with, did it cross with something.

PA No, I think it was as in it was a field experiment, so it was actually at a US agricultural station, a field trial. And it escaped sort of in a puff of wind.

RE Right.

PA Yeah, and that’s quite well documented, it’s on the USDA website. One of their contamination things.

**Reference 8 - 1.13% Coverage**

PA Yeah, I think it, I think there has to be, in terms of scientific risk assessment. There must, it must be a rigorous, a rigorous scientific assessment. And really because, one of the things about GM, it’s like, we saw this very much with, say with the hornless cattle. You know, yay it’s great we have done all of this. Oh, hang on I’ve just came unplugged. It’s just, we’ve just tweaked one gene, just one gene. But one of things is you don’t know what else that gene might have done. Right.

**Reference 9 - 2.06% Coverage**

PA Until it then gets changed bit by bit. So, this is the first bit of changing, is this announcement today, but I don’t think they’re going to change for transgenesis, I think that would be the same.

RE I see, because that was notoriously slow that process. Slow or borderline impossible for a lot of, a lot of businesses.

PA Well again it’s a, that is not cause of the science per se. That’s more because of the societal, you know, people just don’t really sort of want it. So, you know it’s hard to see how that would be different basically. Does that make sense. I mean I don’t think it’s the regulation per se that made it slow.

RE Right ok.

PA I think it was all the sort of objections and so on and so forth that came into it, you know, when it went into council and things like that. It was the sort of political machinations of the EU. So, it’s, again it comes down to sort of society.

**Reference 10 - 0.60% Coverage**

PA But it’s not, it’s not, and this idea of playing God, that’s not irrational, that’s a societal concern. That’s an ethical concern.

RE Yeah, yeah.

PA They may not be able to express it in the science terms, but there’s nothing that’s not valid about that.

**<Files\\Matla> - § 5 references coded [7.69% Coverage]**

**Reference 1 - 0.85% Coverage**

They are really in favour… for the [association] it is a tool that can help to boost plant breeding. It’s really a tool, it is not the aim of everything, it is just like- the idea is to progress in genetic performance to help to have varieties that help the farmers to get better production in the end.

**Reference 2 - 0.32% Coverage**

It is not the key for everything, it is just an additional tool that can really help to achieve those goals faster.

**Reference 3 - 0.30% Coverage**

Just because for them the in vitro mutagenesis was not used before 2001 which is the date of the Directive.

**Reference 4 - 4.08% Coverage**

There are a lot of discussions right now with Ministries of Agriculture, Ministries of Ecology at the European scale and you can see that basically they know that you need to set a new framework some of them may be, agriculture ministries would like to push, you can give easy access to NBT, they are really a tool, and the Commission also say it is a tool to achieve the goals set in the different strategies they have like Farm to Fork or the biodiversity strategies but you have also the Ministries of Ecology that say we need very specific and careful evaluation process so risk assessment to make sure they are safe. So, basically to cease the idea, other Member States and the Commission know we need to change the regulation but some Member States, I would say some Ministries of Ecology say that we need very heavy risk assessment for the agronomy part. We say that it is not necessary to have something that heavy and done for GMO otherwise you kill the opportunity to use it. After we win the debate, we need to have this debate at the European level, we need our scientist and scientific expertise to come to show that if there is any risk to choosing NBT or not and then I hope in the coming 2 years because the goal is to have a new recommendation or a new amendment to the directive in the coming 2 years. This is the goal set by the Commission. I hope, it is quite ambitious because as I told you, it is very sensitive topic.

**Reference 5 - 2.14% Coverage**

The fact that we talk about biodiversity when we are talking about crops, I mean it is not biodiversity, biodiversity is in natural landscape and we need really to work on that, because you have 1,000 more varieties that you are saving biodiversity, I really don’t like but in the Farm to Fork or biodiversity, I have the feeling for them, the regulation could help and it is the wrong debate and with the example of organic varieties but I still have the feeling with NGO’s they ask durability with the technique, what you do with the technique maybe could help but, we ask a lot to techniques to varieties – they will not save the world, they can help, it is a tool to have better agriculture but yes, it is not the key for everything, it is just a tool.

**<Files\\Njavara> - § 7 references coded [10.77% Coverage]**

**Reference 1 - 0.72% Coverage**

So, for at least five years now, we have no new applications and no discussions about new authorisations and things like that. So, we have moved a little bit to other topics. But now, it again becomes very important for us, of course, because of these new techniques. CRISPR and other genome editing things.

**Reference 2 - 1.24% Coverage**

It’s the case it’s always been different. When I look at the whole GE thing and our colleagues in... but the UK has always been a little bit different, where we have not worked so heavily on the topic and, I think, the opinion and the public view on GE has always been a little bit different in the UK. And the politics too. Maybe this makes it now easier for your government to deregulate some things…

**Reference 3 - 1.51% Coverage**

RE I think-, so, the deregulation so far is only around trials. They haven’t really specified paths to market and what that would look like and what kind of regulatory framework is going to be in place. I’d be very surprised if they didn’t, at this point, push for some kind of deregulation. Only for gene-edited crops. But they did say they would look at-, go back to laws around GM. So, first generation transgenic.

PA Yeah. I know. They might be very careful on that because there’s something in the Brexit treaties, I think, that makes it as soon as they lower standards in some topics, then they might run into problems there, so.

**Reference 4 - 2.16% Coverage**

RE So, you said they’re doing other things, as well.

PA Ah. I was thinking about bigger deletions or-,

RE Oh, right.

PA Putting in or adding, as a gain-of-function mutation or a gain-of-function gene. Gene transfer with CRISPR tools or other genome editing techniques to avoid these acquisition effects you have when you don’t do it. When you do it just randomly by particle bombardment or a bacteria. That’s what I was thinking about. And, of course, what you-, I think it’s especially interesting for you when you work on rice or what was the other crop? Potato?

RE Potato.

PA Yeah. Then it would definitely be very difficult to get a homozygous line there. This is not something that you can do easily with genetic engineering, huh? So, to get your mutation into all chromosomes. This is something that would not be possible otherwise, no? Or very, very difficult to get a homozygous potato line.

**Reference 5 - 1.80% Coverage**

And I also understand scientists who say, “Yes, we want to use this and we want to try everything which comes to our minds.” And that’s from the scientific or from an industry point of view, that’s totally okay. But there’s the other view of environmental protection, of consumer protection, of GE-free industry and-, which is very strong in Europe nowadays. And the whole organic sector is being heavily influenced if deregulation will take place because they have decided against using these type of-,

RE Yeah, yeah.

PA Fields [inaudible] and food and-, so, I think the whole decision that should now be made should somehow balance these interests, yeah? What we demand now is just deregulation. So, everybody is free to use these techniques then.

**Reference 6 - 1.96% Coverage**

PA It’s getting more and more difficult because there are no such markers being used now. Or it could be that no markers are being used. This has been very easy. Also, the traits have been very easy because they were all the same in the end. Just different combinations.

RE So, how do you detect-, you know. Do you use-,

PA We do the sequencing out of the site where the mutation has been done but there are other crops that truly have a natural mutation that is in exactly the same position. We sequence out of that and look for differences and then we design the PCR method. That’s pretty simple. And this is, of course, it’s not detecting that ODM has been used in the design of the plan but it’s specifically detecting the line. So, this would be okay as long as it’s specific enough and, yeah. And sensitive enough.

**Reference 7 - 1.39% Coverage**

RE European Union. Yeah, within the European Union. Yeah. So, a lot more funding and attention. It was more a systemic look at what gets funded and why and agroecology tends to-, suffers to attract the amount of funding that other things would. And, of course, that stands in contrast with Farm to Fork Strategy, which wants-, how much is it? 25 percent? Organic?

PA Wow. I don’t-, yeah, I think so.

RE Yeah. By 2030.

PA Yeah. No, that’s certified organic that the US defines some days now, so. So, when we talk about “organic” or “agroecology” that’s more than just what they use.

**<Files\\Ofada> - § 7 references coded [13.21% Coverage]**

**Reference 1 - 1.01% Coverage**

So that would be for us at least the ideal scenario which would also be in line with approaches taken in other parts of the world so it would also allow for international harmonisation which would of course also reduce potential barriers to trade etcetera.

**Reference 2 - 5.00% Coverage**

Yes, it might be perceived like that but to be honest, so this is not at least the intention from our side so it’s actually really a continuum but you have of course different regulatory, let’s say, triggers and for me it doesn’t mean if a GMO a transgenic is regulated it’s a bad thing. It’s just that regulators want to have a second look but in the end the product which will be coming to the market is as safe as a conventional product, otherwise the regulatory system would fail. I mean it’s the intention of a regulatory process to assess if a product is safe which is coming to the market, and if it’s approved it’s safe, but definitely I mean that doesn’t take away that of course also the current regime for GM regulations or transgenic regulation can be improved, definitely. I mean we only have one product on the market in the EU which is this BT corn in Spain and all the other products are let’s say imported but not grown in the EU and so we can definitely improve that as well for transgenics and it should be improved but I think that’s not the focus of the current regulatory discussion which is on targeted mutagenesis and cisgenesis so this is where we are concentrating on but definitely there can be improvements on the classical transgenics as well.

**Reference 3 - 3.19% Coverage**

Yes, I mean for a transgene you have, you need to provide a full dossier of a risk and safety assessment which of course includes data on environmental safety, food safety etcetera with all these kind of rat studies on what you need to do so it’s a really comprehensive overview on all kinds of aspects of safety whereas here, you would say, and I mean EFSA already confirmed this. If a product falls into this category of cisgenesis or targeted mutagenesis the risks are not different from conventional breeding so you don’t need the safety assessment, you just need to confirm if your product indeed falls into this category which is comparable to conventional breeding, also regarding risks, or if it’s different and then maybe more like a GMO or a GMO light or whatever so that you can regulate it as a GMO.

**Reference 4 - 0.16% Coverage**

Well it’s let’s say extensive governance.

**Reference 5 - 1.35% Coverage**

Well I haven’t seen any concrete proposals now, so what they are really going to do but I mean it could be helpful, but I have also the feeling it’s kind of waiting for each other and so the EU somehow looking at the UK, UK looking at EU and so who moves first might be the winner or the loser, I don’t know, so it’s a bit hen and egg I guess.

**Reference 6 - 1.39% Coverage**

Yes, I mean in the EU if you look at… even if you would get approval for growing a GMO you still have the opt-out regulation which allows member states to opt out from cultivation and currently it’s 19 out of 27 member states who had already opted out so there is no market in the end, so it doesn’t justify at all the costs related to product approval.

**Reference 7 - 1.12% Coverage**

I mean if you develop material and you can exchange material or not really do field trials, so I think it’s also a disadvantage for European researchers and so trying to align let’s say, policy approaches around the world in the sense, it makes sense for all different kind of people.

**<Files\\Ondel> - § 6 references coded [19.19% Coverage]**

**Reference 1 - 1.81% Coverage**

You see, I mean, just before I answer the question, I'll explain maybe one thing that there is a different level of involvement and understanding of what NGTs are. There's at the moment we see that only several stations which are actively involved into the discussion. The others are more like in a kind of, on the waiting bench, you know. They're like a more like a witness, just looking what's happening around and that's it.

**Reference 2 - 1.68% Coverage**

How they should be regulated? I mean, it's a difficult question, we are maybe we'll respond like more like a public specialist. We need some clear rules. That's the most important to make sure that there is a clear definition, as I mentioned before that we need to clearly define what plans, which will produce those NGTs are what, how to identify them. And that’s important. Am I understandable?

**Reference 3 - 3.03% Coverage**

Well, we were a bit kind of, because as I said, we're just only starting and getting involved in this topic and we said that we are kind of, confirm the commission's conclusion that NGTs could offer to meet the EU targets for reduction of the use of chemicals. That's the first. We also mentioned, as I mentioned just now that we need a clear definition of what plants are produced by NGTs. And we also mentioned that there should be some procedures to ensure that products that are important on the European market are not NGTs or if they are NGTs then they comply with the EU requirement. Because again, as I explained before, we are looking at this from a trade perspective. We are not looking at the breeders.

**Reference 4 - 2.69% Coverage**

Yeah, but the rules have to be clear. But I mean, we don't have as I say, we don't have a clear opinion, let's say whether there should be separate legislation or whether it should be part of it. We know, we do agree that there should be a revision of the rule, of the current rules. There should be a kind of space given to NGTs basically, in which form or shape it will be given. It's up to regulators to do it because they know it better. But of course it has to be done because we cannot avoid the subject because many third countries, they are really wanting to invest into this technology and we cannot be in Europe behind it.

**Reference 5 - 4.05% Coverage**

Yes, because as I said, we are, I will be because our kind of how do I say, we are dealing with the topic, but the extent we started, the amount of work we did is kind of very limited at the moment. And that's why there will be a lot of repetitions of things which I have said before, because at the moment we don't have like a really strong opinion whether it has to be separate legislation or an amendment to the current directive. Of course we want to have something maybe which is really equally applicable all around the EU and the rules are clear to everyone, that's coming yeah. I'm sure maybe, because we're going to, at the end of this month and maybe more kind of things will come out and we decide on this. Yeah, but at the moment where we are, it's really kind of we at the moment don't have a strong position. But as I said, there is now consultation going and depending on the questions they are asking, we see maybe how to react to that.

**Reference 6 - 5.92% Coverage**

It's not unusual because national stations in many countries they are much more kind of, what is it, they're always in a more waiting mode. It depends on, of course, it depends on National Stations. Some countries are more active, some countries less active. But in general, they are always kind of on the waiting, more innovating more where companies are much more active because it's affected daily business and in our case as well, companies are actually providing a lot of feedback and especially scientific feedback because you have to remember in the national stations there could be more. Depending on the country, there could be just a general, generally, kind of people with general knowledge. They don't have any technical background, and they just say oh it’s too complicated and so. Where other companies, they have resources, and they will be able to assist more in the process because also some national stations, depending on the country that could be one person only or two persons and, well, what can you expect? Because there are a lot of many other issues to be dealt with. And NGTs maybe is not number one priority… if you compare [organisation], it’s like, what eight or nine persons and I mean, that's why you will have, how to say, more details from them than from us. Because I deal with many, many topics.

**<Files\\Patna> - § 9 references coded [10.45% Coverage]**

**Reference 1 - 0.58% Coverage**

… see this one’s really quite interesting because patenting is a big part of it and there are a few voices opposing patenting on the GM side at the moment and it, now that’s partly because some, there are some advocates for complete free rein, like zero regulation whatsoever. Let people do gene editing in their shed, little bit scary.

**Reference 2 - 0.98% Coverage**

But, you know, the patenting regime for gene editing is already largely owned by Corteva who, which who came back from was it DowDuPont and one of the others, came together to and I don’t know if it’s Corteva or Corteva, probably depends where you live in the world. Corteva even. You know, these what happens is even when it’s new, so the universities have these little offshoot companies and they’re like, oh, we’re a start-up, we’re really nice and we’re not big corporates and they do the research, and they lodge all the patents but then they get bought out.

**Reference 3 - 0.86% Coverage**

Well you can if that’s all you’ve done but there’s always this, like, they always talk about the part of the process that does the thing that the point of the project is, but actually, the do a lot of other things along the way. So, you know, it’s all very well saying oh, we can just check for the thing we’ve added but you haven’t just added the thing that you’ve added. You’ve added a load of code either side of it, you’ve added a load of reagents to cause it to be entered into the cell.

**Reference 4 - 1.01% Coverage**

I would improve the independence and I would probably have repeat risk assessment on maybe something like a, you know, a ten-year cycle so that you can actually look at what has happened once the thing’s been approved. But, you know, somebody other than the research team checking what’s happened, I think you absolutely have to have [unclear]. If you’re going to release novel genetic constructs, you know, whether you’ve added something in or you’ve amended what was there, you’re still creating novel DNA and you’re releasing that into the environment, you need to monitor it.

**References 5-6 - 1.45% Coverage**

You need traceability all the way along the food chain and then if something goes wrong, you can record it and do you know, there is huge consumer demand for labelling. People want labelling because there are, you know, this broad range of issues with the application for technology and people want to be able to avoid it. So that’s what we get from the current regs. We could all rewrite them, we could all say what our perfect thing would be. I would like to see much greater assessment of the social and ethical impacts you know, and I would really like to bring in trait impact assessments as well as process. But it’s not a trait or process, it’s both, you know, the way that you’ve changed something brings about one set of risks. The thing that you’ve ended up with, proposes another set of risks and both should be assessed.

**Reference 7 - 1.55% Coverage**

I mean journalists just repeat all sorts of extraordinary things, so you know, there is definitely kind of a fiction out there that the rules always said that GM was transgenesis and then the ECJ made this strange decision. Now the ECJ spent two years studying the science. They’ve done the most in-depth consideration of this of anybody in the entire world and they came to the entirely logical conclusion that of course, the description in the EU regulations includes genome editing. It also includes radiation mutagenesis. It also includes chemical mutagenesis. It includes everything that basically doesn’t involve sex and then for political reasons, exemptions were made for certain techniques that were already widely used. That doesn’t mean that everyone loves those techniques. It means that a political decision was made to allow them or to allow them to escape the regulation.

**Reference 8 - 1.04% Coverage**

PA But it’s not just DEFRA, there is a much broader context of deregulation. So if you’ve looked at the BEIS consultation that just closed quite recently, okay so do you know the TIGR Report?

RE Yes.

PA Isn’t it a delight? So coming out, one of the things that’s come out of that is there’s so Bayes had a consultation on reforming the framework for better regulation. So, yeah, it’s kind of like three steps down the, or up the line, up the hierarchy in that, they were being consulted on how they should reform the framework that they use in order to consider reforming the regulation.

**Reference 9 - 2.97% Coverage**

Obviously it’s come from a very particular perspective. But, that stuff’s all going on in the background so there is, I think, yes, well, I mean Boris Johnson mentions, what was the phrase he used, he was going to liberate us, liberate our extraordinary bioscience sector from the restrictions of GM rules. That isn’t quite, oh from anti-genetic modification regulation, that was it and I think there’s, so there’s a political agenda from the top that I don’t think has got like, it’s not really got anything to do with GM, what it’s got to do with is general deregulation. So sort of, you know, making things easier for industry and there’s no public support for that whatsoever. There was absolutely no evidence of public desire to reduce regulation and lots of evidence for the public support of strong protections. So, there’s that kind of agenda and that’s very much where the BEIS stuff and the TIGR Report all come in. But I think there’s also, that phrase, liberate our extraordinary bioscience sector, so that’s about the UK is good at something technologically and we need to be more good at, you know we need to have more things that we’re good at that make money for the country. There are restrictions around that, that might be somehow limiting how much money I think we can make. Do you know, so it’s not I don’t think Johnson comes at it from an understanding of the technology at all. I think you know, he’s been told, we’ve got all these really, really clever scientists, you know, but we’re not making millions out of it, what can we do. I think there is a massive corporate lobby involved in all of this.

**<Files\\Rufipogon> - § 8 references coded [15.52% Coverage]**

**Reference 1 - 2.39% Coverage**

… if you want to build, if the regulator and then it also depends on what we’re talking about in terms of regulation, so in the European Food Safety Authority which is obviously the key agency in this space, already has guidelines for risk assessment in GM animals and for GM crops. So what they… their role is to present scientific risk assessment frameworks and they do that through guidelines. Those guidelines then dictate to applicants, what process to follow and what evidence to supply etcetera and then it’s reviewed by us, individual applications, so there’s two stages in risk assessment. One is the actual risk assessment itself of the individual products but the other is establishing the guidelines of the framework, so as the guidelines… they’re also sometimes called risk assessment policies because they hold them because they set the framework for risk assessment, they have the policy kind of questions where they have value-based questions. They’re not purely technical.

RE Okay.

PA So then the commissioner is the risk manager in Europe.

**Reference 2 - 1.03% Coverage**

It’s not quite sure what that’s going to look like in the UK yet but we have ACRE the advisory committee on releases to the environment which is the equivalent of ESA and we expect that they will take a lot of these GM crop applications. Whether they set up guidelines is not clear yet.

RE So how would they operate if they didn’t have guidelines.

PA Well they may adopt EFSA’s.

RE Right, just take it wholesale then.

PA They may do that yeah.

**Reference 3 - 5.20% Coverage**

And there are people, and the components and the funders do want it so what happens is risk assessment becomes this highly politicised space and there are efforts to keep that scientific by those who benefit from keeping it scientific and there’s an effort to politicise those by people who want to have that political suppression, so I think that’s the most important thing is that regulations are never neutral and claim that it could be science based is just a fallacy.

RE Also, I mean is there, should it only be science based if that is a thing and it can be, should it be anyway yeah. There’s always some value judgement.

PA Yeah, so who you choose on your expert working group makes a difference. So at the moment we see in the GM so EFSA’s just provided guidelines to order, a report which was assessing the adequacy of guidelines for gene drive organisms whether that’s agriculture, environment or whatever, so they assessed the GM animals guidelines to decide whether they were adequate for gene drive and the conclusion was that mostly they were but there were some changes needed. But that’s, if you look at who’s on the working group there, they are predominantly developers, people who have worked in either developing gene drive or worked closely with developers of gene drive… they have a certain view of risk and of the value of biotech and science. So you can say it’s science based but it’s science based in their view of the science and if we brought in ecologists they might be the best Nobel laureate ecologists in the world and you bring them in… they’re going to have a different view of this so nothing to do with a degree of expertise, it’s to do with the way they understand science, so recognising that regulations and risk assessment are political spaces is the first important thing, so whether you want it science based or not is not a question for me.

RE Is it fair to say that the word science, like, oh let’s have science-based risk assessment is usually used as a kind of a blunt tool to disarm like opposition groups from saying you’re about science.

PA It’s to exclude.

**Reference 4 - 0.63% Coverage**

… the proponents of GM crops in my mind just don’t handle it well. The disregard for the full range of issues which need to be examined and this trying to exclude those issues and focus on whether it’s safe or not is not going to win people over. It’s not going to win people over.

**Reference 5 - 1.01% Coverage**

We know what they care about, so one of the key issues which I’ve focussed on, so issues around naturalness is not my space, but that’s obviously something, that’s the hardest nut to crack I think. Issues with governance we can do that, right, and the questions you’re asking about risk assessment and regulation are key to that alright. So what we need to do is built legitimacy in those institutions, that would address the issue of public trust.

**Reference 6 - 0.93% Coverage**

So the idea of seeing science as less exclusionary is an important step moving forward. Now, NGOs are interest groups and so I do think that there’s a way of moving this debate forward is this removal of focus on interests and stakes which doesn’t seem to be getting us anywhere and all that happens is that the developers want to push that debate into the scientific risk assessment space which we can't help it…

**Reference 7 - 3.36% Coverage**

Good to go yeah. And the failure to label in the US I think has been really detrimental for decades to come. This idea because the premise there is that in the US and Canada is that you don’t need to label it, so it was that you don’t need, you have risk assessment and then it’s safe and then people can make the choice of whether they want to eat it or buy it or support it based on their own values. So we have this kind of value-free, scientific risk assessment process and then the value decisions are made by individuals further downstream. But of course you can't make those decisions without labelling, so if you’re going to take that argument that you keep risk assessments as technical as possible and allow the value decisions elsewhere you have to give people the power to make those decisions so the labelling issue in the US, you had a really undermined legitimacy of that rationale for science based risk assessment. Now we don’t have it here, like we don’t have GM any more. A few tiny pockets of things in Spain and other stuff but I mean, really, I can't say we have GM here when you think that in the US it’s been grown there since 1996. We can't really say we’ve got any progress here in that sense, but the kind of, you need to have this debate, this democratic debate about whether we use them or not needs to be had somewhere. The government’s recent consultation around gene editing in the UK just, you know, the language used is clearly showing that this is what they want.

**Reference 8 - 0.97% Coverage**

PA So that construction I find quite interesting, that’s something that’s been constructed at the moment, the language around gene editing and GM.

RE They’re trying to reject that distinction, a few years ago that was really important because gene editing was natural.

PA Oh yes, because if they can show that it’s different and get the regulations into genome editing and then say it’s the same, they can pull GM into that space.

**<Files\\Salari> - § 8 references coded [22.51% Coverage]**

**Reference 1 - 4.49% Coverage**

As I said, the organic movement is diverse, but a very big opposition towards genetic engineering comes also from corporate control. And what do I mean by this? It's because, if you look at the patent law, clearly it is a technical intervention. This means that you can patent it, you cannot only patent the process by itself, but you can also patent the modification event. To put it correctly, you cannot patent a plant, but you can patent an event. This means that those, let's say more classical breeders, those who are part of having founded this organic movement, they would be restricted in the kind of plant material they can use for breeding. Also because, I don't know if you know, but if you have multiple plants and you modify them in a way that they all have, for example, resistance against Phytophthora, then you have kind of a patent on a plant trait. Then if you replicate that with classical breeding, you can make a claim on the specific trait. So for organic, it is perceived as a danger. Also, because being able to patent plant traits, you can also, let's say limit the space for classical breeders. So this fear about corporate control over the seed world is a very big reason also for this opposition.

**Reference 2 - 3.64% Coverage**

The organic movement has a very clear position on this and how it should be regulated. This is that we have a regulation right now that is kind of working because of the three aspects, which is you have a risk assessment, you have traceability and labelling. So from a value basis, organic also very much comes from this precautionary principle that this is a technical intervention, this might have implications that are beyond the capability of scientists to fully grasp which is why organic sees itself going more with nature rather than against. So it kind of flows clearly from that, that organic would not be, let's say, an early adopter of these techniques. Because of these potential environmental implications, it's very important for organic that there is a risk assessment that looks, of course, also at humans, at health for humans, but also at the environmental perspective, other interactions, for example, with insects or other plants, so this risk assessment is a strong point.

**Reference 3 - 3.10% Coverage**

Then the biggest point probably is the traceability, which means that you have a full transparency throughout the whole food chain to see which products are used, which seed has been used and how this seed has been produced. This is, on the one hand, important for farmers to be able to not use GMOs and also important for breeders in order to not use GMOs in their breeding processes and it's very much also connected to consumer expectations that there is a certain group of organic consumers that do expect organic to be GMO free. For them, it's also a big reason to buy organic, and the only way to guarantee that is traceability. This can be done in different ways. It can be done via e-traceability, or you can have these detection laboratories that test imports on the presence of certain organisms. So, traceability is the biggest point.

**Reference 4 - 0.85% Coverage**

It's a big issue because, we had this a lot, that they are not detectable. Actually, the organic movement has been part of financing a project that has developed the detection method to detect one of the genes edited, I think canola.

**Reference 5 - 2.19% Coverage**

So it is possible to develop detection methods also for gene editing approaches. Of course, it might not be possible in 100% of the cases, but here we have more of a pragmatic approach. You say, if we know about the plant, if we know it's on the market, let's develop the detection methods, and as with current GMOs, you can never be sure. If someone develops a GMO, doesn't register it, just puts it on the market in the US, exports it to the EU, you would also not be able to find it, so it might not be possible in 100% of the cases, but in the most relevant cases, it seems like it would be.

**Reference 6 - 5.09% Coverage**

I can only speak for what we are doing and what I am doing, and the risk assessment indeed should look at implications for human health. You can do studies regarding allergens and so on, and you can also look at the environment. You can completely say that how a plant interacts with a certain pollinator, you would have to do a field trial for that probably, but of course, this is scientific. I think what many groups are saying, what we are also saying, is that you cannot look only at the trait in isolation, but you have to see it in combination with the application on the field. So if you do have a herbicide resistant plant, you have to analyse the impact on environment in combination with the pesticide that is also likely to be used. But they are also still in a scientific realm, and from our perspective, everything that goes beyond that, let's say, questions of ethical concerns like values, this aspect of corporate control over the seed market, this is more captured by the traceability and by the labelling because it gives a certain freedom to choose for something or against it on the free market. As I said, for some consumers, this aspect of something being free of this technical intervention is something that they value. So, I would say that risk assessment has to be, by definition, kind of done in a scientific way. There is no other way to do a risk assessment.

**Reference 7 - 1.47% Coverage**

Yes, I think you raise a good point. For example, if you look at intervention in humans and embryos, where would you capture it? Would you capture it in the risk assessment, because traceability and labelling is good, but there are ethical questions? The question is, where would you put them? Would you put them in the risk assessment? That's a good question. I don't have an answer to it right now.

**Reference 8 - 1.67% Coverage**

I just think in general, if you want to start, if you talk about biotechnologies, it is crucial to, not only look at the technique, not to only look at how is this certain technical intervention more or less invasive, how is it replicating potential processes happening in nature? This has to be looked at, as well, but it's always important to look at the economics behind it, at the ethics behind it, in order to be fully able to grasp what is happening.

**<Files\\Sharbati> - § 7 references coded [14.78% Coverage]**

**Reference 1 - 0.94% Coverage**

But, really, to have a proof that is law safe is hard to do. Especially when you have outcrossed the tools for genome editing, then detection and identification might be a problem that will pop up in the coming months, even.

**Reference 2 - 2.07% Coverage**

I mean, currently we have only two genome-edited crops, or one real genome-editing crop on the market, which might enter Europe. This is the Calyxt soybean. And with the highGABA tomato from Japan, we have another coming. But Europe has the advantage here that they are both identity-preserved products. So, especially, the soy bean is coming in a circle environment. So, the company is selling the seeds and buying everything back and selling the oil. So, it’s never an open commodity shipment.

**Reference 3 - 3.25% Coverage**

I say, the only market is the US currently. I think they are doing it because it’s a good business model and they get higher prices if they do an identity preserved. It’s the same in Europe with the, for example, the pink lady apple which is also an identity-preserved product, which is never in the open market. I think, when the waxy corn from DuPont is coming, the European Union will face more severe problems in detection of it. And, we don’t know which other products will pop up in the coming months and years. I just saw a paper from the colleagues in the US, from Neil Hoffman, concerning the revision of the SECURE rule that they have currently. Let me check, 168 non-regulated status products. So-, and we don’t know about what will come because they are secured and-

**Reference 4 - 0.56% Coverage**

RE Right. So, the regulation in Europe hasn’t yet been tested to a-,

PA Right. And I think it will be tested in the coming months.

**Reference 5 - 2.17% Coverage**

RE So, what are the prospects for being able to detect a genome-edited crop, versus any other?

PA There are quite a lot. As long as you have the tools still in the crop, it’s not a big problem. Because then it’s just a detection as with classic GMO, because you can do a PCR primary, for example, on the Cas enzyme and you’re fine. But, as you have found a point you have outcrossed the tools, then you just stay with mutation. You can identify the product but you can’t detect if it’s a genome-editing event or not.

**Reference 6 - 5.17% Coverage**

PA It’s impossible. Technically impossible.

RE Is there something ironic here about the fact that if it were a GMO, it would be detectable, but GMOs are very unacceptable to certain groups-,

PA Right.

RE: In the European Union? That seems a bit-, when I say, interesting, I didn’t know that. That-, yeah, I didn’t realise you could leave behind the tools, let’s say. I knew you could outcross them, but I didn’t know there was-, usually they’re outcrossed, right? Because they’re-,

PA Ah, yes. But you can also do it completely without integrating the tools into the genome in the first place. So, what we are doing at the moment is that we are just using the isolated Cas9 enzyme in a combination with the guide RNAs, the so-called r-gene complex, the RNA-guided nuclease complex, and bring them in as a protein. For example, in protoplasts or in somatic embryos-,

RE Right.

PA And they cut and they are dissolved by the cells in 72 hours and they are gone, so you don’t have any trace of editing and have just imitation. I have never used recombinant DNA because it’s just a protein and an RA which has then gone and you just stay with the mutation. And there is no chance of integrating any foreign DNA into the plant.

**Reference 7 - 0.62% Coverage**

And this is said the Parliament can push the Commission to give up a statement or initiate a policy action. And so the communication ways currently.

**Name:** Key actors and pathways to influence

**<Files\\Ambemore> - § 3 references coded [3.00% Coverage]**

[redacted reference]

[redacted reference]

**Reference 3 - 1.52% Coverage**

The Commission wants to make the consensus amongst the society and in the other hand the Commission reflect how much chance they have that their proposal passed through the inter-institution negotiation – if they don’t feel they will be supported by the Member States or by the Parliament, they take their seat but they will not move and yes, of course they have allocate their resources to some priorities and to push on initiatives that has a chance to succeed.

**<Files\\Chandra> - § 1 reference coded [1.56% Coverage]**

**Reference 1 - 1.56% Coverage**

Yeah, so in terms of the bill, we don't believe that it's very likely that the bill will be amended through the different stages that it goes through Parliament just because of the way that the government is set up right now, it does seem very unlikely. So our focus is kind of making sure that some of the issues that I've discussed are at least aired and get better understanding because a lot of it we're going to secondary legislation and it would be, you know, it's our hope that even if some of these amendments don't go through in terms of the actual bill itself, that they will be taken into account with secondary legislation. So for example, that at the moment the bill is phrased such that traceability is a may rather than must, and so ideally in the secondary legislation there would be a lot more looking into why traceability is necessary from a business point of view.

**<Files\\Ginpu> - § 3 references coded [7.01% Coverage]**

**Reference 1 - 1.16% Coverage**

In the UK, I guess the National Farmers’ Union England would have been very influential in the past, probably less so now with the current government that we've got.

**Reference 2 - 3.28% Coverage**

I think so, yes. I think this a fairly recent change. I think environmental NGOs are becoming increasingly influential and then you'll have the marketplace, supermarkets and retailers are probably the most influential. One of the issues is seed potato exports, big problem. You can’t export to the EU; all sorts of problems anyway. McDonald's have lobbied to allow EU imports to come in and McDonald's got their way whereas [farming organisations] didn't get their way.

**Reference 3 - 2.57% Coverage**

I would say that increasingly we're more towards big businesses that are more influential. It’s quite different in [country]. I would say that [farming organisation] are very influential in [country] Government and other membership organisations and then environmental NGOs as well and probably industry, the market being a bit lower down than the rest.

**<Files\\Illabong> - § 3 references coded [5.43% Coverage]**

**Reference 1 - 0.95% Coverage**

PA You don’t have the National Competent Authorities. All the Member States if they want to get something for approval they have to go via National Competent Authority.

**Reference 2 - 1.91% Coverage**

This is definitely the non-governmental organisations. They have a lot of impact on the decision making processes. French and German NGO’s are definitely the most influential or they are the most vocal ones. Maybe they have the deepest pockets. I think it goes hand-in-hand with the size of the country as well. NGO’s are more powerful.

**Reference 3 - 2.57% Coverage**

I would also look into how consumers look into it and also the supply chain like retailers and food processors may look at it. They may not only consider the UK market but it is specific to the areas of the crop you are talking about. If you have exposure to a large international market then for those in the supply chain also for what other countries are doing will become relevant and if you export to the EU for example you may want to consider twice.

**<Files\\Jata> - § 1 reference coded [1.64% Coverage]**

**Reference 1 - 1.64% Coverage**

Yeah, I think it’s not about who is right or wrong, but I think it’s about indeed, communicating about where the science is right now towards the family, towards you know, friends, towards anyone else so that people get a better understanding actually of what’s happening and to where this technology if you talk about genome editing, is right now.

**<Files\\Kebo> - § 3 references coded [3.69% Coverage]**

**Reference 1 - 0.96% Coverage**

RE Absolutely. What organisations or entities in the UK do you think are most influential in determining policy in this space?

PA DEFRA.

RE That’s where the buck stops. Who influences DEFRA do you think? Who has that ear for want of a better phrase.

PA They pay attention to the Royal Society but they certainly don’t listen to everything we say.

[redacted reference]

**Reference 3 - 1.00% Coverage**

So who are the other players? Of course the supermarkets will be taking the view the second mouse gets the cheese. They’re all terrified about damage to their precious brands if they’re a first mover and then they get crucified. No supermarkets want to take the risk of having [NGO]-led demonstrations outside their supermarkets against GM [crops] or whatever.

**<Files\\Koshihikari> - § 1 reference coded [2.49% Coverage]**

**Reference 1 - 2.49% Coverage**

RE Which organisations or entities, if you want to call it that, do you think have the most influence over these decisions. About regulation?

PA That’s interesting because if you had asked me 10 years ago, I would have said your Monsanto, your Syngenta et al. Now I would actually say much more sort of public research institute scientists.

RE Like, like thinktanks or well-known scientists, that sort of thing?

PA The Rosalind institute. They’re there. British society of plant breeders. You know those are two that are on the governments press release.

RE Right, I see ok.

PA So, they must have some influence so these are the sorts of things they are getting in, and I think, I think what’s happened is you know scientists are thinking, oh great, you know we can do this, we can have, you know cows with no horns. You know we can have, they, it’s a bit like the early days of the first generation when it was going to be, oh we can do all these things. Do all these things. And you couldn’t. Actually, all you could do was herbicide tolerance and insect resistance.

**<Files\\Matla> - § 4 references coded [7.56% Coverage]**

**Reference 1 - 1.36% Coverage**

I mean, as you say it is not really the size but as well I see that maybe I’m wrong here but in fact I do not know if the Member States or the blue circles should be there because in fact they are really the one that do the policy and in fact they chat with NGO’s, with industry, society and then translate what they understood, what they got, what they would like to understand also and also to make the policy. So, for me they are not really in the same circle as the stakeholders.

**Reference 2 - 1.40% Coverage**

If you show that to someone in the street they would say ok, in fact Member State or industry it is the same. In fact, industry or NGO or so, they do not modify the legislation at the same time or after the Council or the Parliament, they do that before in fact you see. So, for me, they are more a filter the European Commission and also the MEP and they try sum up, to synthesise what the stakeholders think up, sometimes maybe NGO or sometimes industry would lead that would have more impact.

**Reference 3 - 0.44% Coverage**

RE I see. On this topic as well, who do you think has the most influence, I think that you’ve alluded to it there.

PA To be honest, I think we don’t know.

**Reference 4 - 4.36% Coverage**

Who had the most impact but at that time now, it is impossible to say, [industry association] or [NGO] will have more impact on the Commission. It is just impossible to say – maybe and I would like that the Commission takes it’s time to understand to listen to everybody obviously but they are so aware of the importance – maybe it should be 95% of the panel, 19,000 comments on the consultation, maybe you cannot really take them into account and you may speak with people that know a bit, scientists, stakeholders like for instance, say the industry, seed breeders, people that are interested in the theme but maybe the point of view should be based on science and not on beliefs – the role of the Council, or the Parliament and the European Commission is to represent European citizens, it is just that some times, it is easy to manipulate the option of the European citizens. So, I do believe that sometimes it is better to ask experts even if it is difficult during the past few years to give credit to experts because the people come to say no, experts are not good, they are biased and they are corrupted and so and so. At least at [organisation] and I know that the Ministry would like to put science first and after you need many years to explain why science says that it is safe, or you can use that, or CRISPR is different than transgenic. It takes a lot of time and effort and cautious, so it is very difficult time because so many people sometimes just try to spoil the debate and then you cannot say anything and afterwards it is just…

**<Files\\Njavara> - § 6 references coded [5.87% Coverage]**

[redacted reference]

**Reference 2 - 0.23% Coverage**

And, yeah. I hope that public opinion-, and we can play a role in that. So, yeah. And we’ll see.

[redacted reference]

**Reference 4 - 0.46% Coverage**

PA But, of course, there are also NGOs that are promoting biotechnology, no? So…

RE Yeah. Definitely.

PA Just say non-governmental organisations there are some that are very much in favour.

**Reference 5 - 0.79% Coverage**

Then you might want to put other states or other-, along here. Round here. Because I think Brazil or US, they have big interest in-, this is international trade, of course. But we know that they have been heavily lobbying for GE in the past in [country] and in Europe, so. Because these are export markets for them, no? So, they will be-

**Reference 6 - 1.34% Coverage**

Yes, they will. They definitely do. So, we are really-, and, so far, we depend on imports from North and South America when it comes to soy, for instance. And the US has always been very friendly in authorising imports but, yes. This might become more difficult and that’s why deregulation, of course, would be the easiest way for industry or for the big trade organisations. So, I don’t think that they are here in this tree? International trade, yeah. Okay. You know this ABCD complex? How’s it often called? These are the big traders, ADM, Bunge, Cargill and Dreyfus.

**<Files\\Ofada> - § 3 references coded [2.29% Coverage]**

**Reference 1 - 0.69% Coverage**

I think from the national competent authorities there were also others besides BVL who contributed, for example, the Dutch I guess as well as the Swedish Board of Agriculture.

**Reference 2 - 0.42% Coverage**

EFSA definitely I’d say. Ah, maybe what is… no, you have member states which is Council actually I guess.

**Reference 3 - 1.18% Coverage**

I guess I would make politicians and MEPs in blue as well because they belong to the same kind of group I’d say. EFSA, well I’m not sure, I mean they are linked to the European Commission as well, like the JLC for example. I'm not sure if, I think I would move them around this European Commission.

**<Files\\Ondel> - § 2 references coded [7.05% Coverage]**

**Reference 1 - 4.31% Coverage**

That's what they said during the meeting, publicly. And, but you see, maybe also at this stage, it's kind of, it's very limited to how you, who can influence the commission, because at the moment they have to be, they should be based on, if their proposal or whatever they will come up with, they need to base on their signs, and that's kind of my opinion. But next, of course, next step, once it all will go to the Parliament and the Council. Then of course, politicians will play control, and there will be people will scream louder to politicians, either consumers, either NGOs or industry and yeah. I can tell you many, everyone talks about transforming European agriculture and to make it more sustainable. You have to do it somehow with something. You cannot just, how to say, plant vegetables and water them only with water and then expect that you will have a high yield and you will be able to continue feeding the increasing population, the world population only on water and you need something to help.

**Reference 2 - 2.74% Coverage**

And another thing. Member states, the Council of the European Union and European Commission. I would divide this into two categories of member states or, because you see, how things, how European initiative process works. Council of course, it's all composed of member states and the council, and the governments are directly represented there, and they're the ones actually that determine it. And also member states, where they also influence the discussions in the European Commission as well. Okay, maybe keep it separate, doesn't matter. But I would kind of put them maybe council commission and member states to get three circles together.

**<Files\\Patna> - § 3 references coded [2.90% Coverage]**

**Reference 1 - 0.60% Coverage**

RE Who do you think has the most influence then, I might be able to guess but who has the most influence over the process of making regulations in this sphere, who do you think?

PA Well I mean it’s the Secretary of State who signs it all off. I mean civil servants obviously contribute detail, but I think in DEFRA, it is very minister led.

**Reference 2 - 1.42% Coverage**

PA Yeah so they, they operate in a way that is very different from most APPG’s. They are funded, very heavily funded by industry bodies.

RE Right okay.

PA So they’ve all got nice names, but you’ll probably be familiar with the nice, they sound like nice names and like, you know they plant this, but they are funded. I think their funding is relatively new, the group’s been around for a long time, but they didn’t really do anything for a long time, so I think they’ve got, you know they’ve been, investment has come their way to make some noise on this front. Now they were involved with an amendment to the agriculture bill last August which was a really strange thing. It was an amendment to the Agriculture Bill in the House of Lords and very few amendments in the House of Lords ever get taken up.

**Reference 3 - 0.88% Coverage**

So the Nuffield Council Bioethics have been looking at, now unfortunately they’re only looking at animals which I think does skew the way it works. But they’ve had, they’ve been considering as an area of study, genome editing of animals for several years and they invited written evidence and they’ve had various stages in various different ways and they recently did, now what they call a rapid online dialogue, so it was not what they really wanted to do. They wanted to do something much more in depth.

**<Files\\Rufipogon> - § 6 references coded [7.23% Coverage]**

[redacted reference]

**Reference 2 - 0.57% Coverage**

So research wise it’s definitely obviously the two Rothamstead stations, and Roslin, those are the big agricultural research centres. They do have power, they see themselves as the kind of victims of higher research decision making, but they have power.

**Reference 3 - 1.16% Coverage**

Universities have power too, so universities I think could be doing a lot more. We’re always consulted, as universities we’re consulting on the direction of research. We get some leeway to shape calls by what we submit to them. So we definitely have, we are empowered in certain ways. We could think about how universities engage with those stakeholders more broadly so we could be doing a better job of integrating social science into undergrad programmes, having interdisciplinary centres for doctorate programmes.

**Reference 4 - 0.73% Coverage**

Oh and then you’ve obviously got industry. I mean well, so I think it’s not even a “who” right but we know the UK economic policy coming out of base is about economic development so plant biotech, from the government’s perspective or from DEFRA and a basal perspective I would say BEIS is a bigger player than DEFRA in this.

**Reference 5 - 2.61% Coverage**

In my mind BEIS has more power than DEFRA. DEFRA is a weaker institution compared to BEIS. So BEIS sees plant biotech as a contributor to economic growth and international competitiveness, that’s it. It doesn’t see any interest in solving some of the challenges that we’re facing. DEFRA probably does. DEFRA will and the farming, so the NFU, other farming representative bodies, they will see some soil association, others definitely see some, see plant biotech as needing to deliver these other values. So if it only serves economic value and doesn’t serve these other values, then that determines which players voices are most able to influence, so I’d say at the moment it’s obviously on the research side you’ve got the universities, the research councils, again research councils are huge but they’re obviously run by BEIS. They sit under BEIS, so the power BEIS has is enormous. It sets the research agenda. DEFRA does not. DEFRA is powerless in this space. It can run the consultation but it’s being told to do that, there’s no question about that. By the ministers, by the government ministers, right so this government in particular who tries to see that.

**Reference 6 - 0.74% Coverage**

And the House of Lords, also quite powerful in this space. They’ve written a report, they’ve written a few key reports. They wrote a key report on GM insects.

RE Yes, yes.

PA And they wrote the report Science for Society, that’s their report too and having given evidence to the Lords, the Lords are big landowners right.

**<Files\\Sharbati> - § 6 references coded [7.10% Coverage]**

**Reference 1 - 2.42% Coverage**

Ah, currently it’s definitely the European Greens, which are doing a lot here. As a political organisation and as NGOs, all the associated ones, like [NGOs], which are really pushing against this new regulation and really are on the track that all GMO needs the same regulatory oversight, or even stricter regulatory oversight. So, I think they are really active, currently. And the scientific organisations are always a little bit behind. So, they try to motivate their members to get involved but it’s always for science they sometimes come a little bit slow.

**Reference 2 - 0.41% Coverage**

PA What about the Scientific Advice Mechanism from the European Union?

RE Ah, that’s good. Yeah.

**Reference 3 - 1.26% Coverage**

PA And the JRC, the joint research centre. Because they also provided an opinion, I think, in April.

RE Yes. Yes, I think they did. What was the name of the first one you said?

PA Scientific Advice Mechanism. They provided an opinion in 2018, comparing all the different techniques. It’s the SAM.

**Reference 4 - 0.66% Coverage**

PA Perhaps you could add in international trade. So, the World Trade Organisation.

RE Yeah. Who have their own-,

PA I think they also made a statement.

**Reference 5 - 1.10% Coverage**

And as trading organisations are using their power and-, in not accepting genome-edited products, which are obviously not on the market, but they say, “Okay, we won’t put them on our shelves.” And this is forcing, then, different other stakeholders to react to it.

**Reference 6 - 1.25% Coverage**

Milling industry. But if I think of the EU, German market, I know that, for example, Aldi and Lidl, and the Inapa Group all said that they don’t want genome-edited products in the market because the consumers don’t want it and their store’s been forced by the Greens. And this is building pressure.

**Name:** Risks and benefits of biotechnology

**<Files\\Ambemore> - § 10 references coded [14.91% Coverage]**

**Reference 1 - 0.33% Coverage**

… they see risk for themselves and they see risk for the food system and for the consumer at the end.

**Reference 2 - 2.30% Coverage**

… because at the end the issue that we have, the farmers they are exposed to climate change, with the heat, with excessive water, they have huge pressure because the Commission want to escape conventional plant protection product and in some crop we have really a challenge to protect the crop it’s mainly in rape seed but it is also in sugar beet and then farmers need really to have better varieties that resist fungi or to pests. What we need is to accelerate the natural process of breeding and new breeding techniques could do that and by postponing always the solution at the end the farmers pay the bill because the consumer does not really pay the bill, what is not from the EU is from abroad.

**Reference 3 - 1.66% Coverage**

The fact that it is a new breeding technique then we believe that we have also seen in cereals that outside Europe the yields are increasing and the gap in the yield has been reduced because we do not gain any additional productivity so quick as in the past and then really the farmers need improved varieties, well documented that perform much better and at the same time we need to keep local breeders because, local breeders can sell varieties much well-adapted to the local climate and soil conditions.

**Reference 4 - 2.36% Coverage**

I think also- and I lack expertise, new genomic technique has been used to develop the vaccine, I have heard about it. It has been accepted by the population that to develop the vaccine there is no debate, but for agriculture there is a debate. Then maybe we could find some link on this because, I have heard some time this kind of argument. Some techniques, when they are used to save lives, citizens don’t think about it but, if it is about food, it becomes a big issue, and nobody wants it. I’m wondering if we maybe could find a way to develop a kind of communication that explains what we do for food or for crop is the same as we have done for the vaccine. It is accepted because people take care of their life.

**Reference 5 - 0.22% Coverage**

Don’t you think that we could push to have some positive influence.

**Reference 6 - 0.58% Coverage**

It is also understandable- but I think your point is correct as long as people does not feel a risk that they will have a lack of food, the mentalities will remain as they are.

**Reference 7 - 1.56% Coverage**

Then the Commission has also to look at the mainstream because, for example most people in [organisation] believe that they need to have access to new varieties because the constraints to produce organic are much higher compared to conventional, even if the constraints are reduced and if the Commission want to reserve 25% of the land to organic production there is also a need to produce more because if the border closed we will have a lack of food or lack of raw material.

**Reference 8 - 4.73% Coverage**

You know, it depends on the climatic condition but, if there is a huge variation in the climate and if it falls, risk on the crop, sometimes it can huge crop or less crop but when you have less crop may be the borders are not so open as today because look today in the cereals market, the Russians to keep the level of inflation in Russia they play with export quota or export taxes. You have the same with the Ukrainian, we have in Morocco depending sometimes they have an import duty to import cereal sometimes they decide to relieve this import duty. Then Europe has not since many years, since the 90s play with the Common Agricultural Policy and the Market Protection and the Market Management – but at the same time we have signed so many bilateral agreements that finally we can take from abroad a lot of products, but they have also [unclear] and they can decide to not open their borders to feed Europe. Then, I think the Commission can also improve its communication. The Commission as communicate… can do a lot we have seen the CAP when it was created after the Second World War, we see how it has deliver after they had changed the model. Until now, consumers are very happy because over 50 years and more I think the consumer has never depend so less for food because consumer can spend a lot of money on plenty of un-useful and not indispensable goods – yes consumers, they take it for granted but until when. We don’t know.

**Reference 9 - 0.62% Coverage**

Well, what will be the consequences of not regulating new breeding techniques for the European citizen because again, generally speaking everybody takes the food supply for granted but, yes.

**Reference 10 - 0.55% Coverage**

We know that in Europe we are not good in this sector because of lack of productivity – could have aspirational target to improve our productivity on this kind of crop.

**<Files\\Chandra> - § 17 references coded [16.71% Coverage]**

**Reference 1 - 0.92% Coverage**

So that's a massive, massive shift that we need to do and involves huge, huge, huge amounts of changes that are, and like technological change yes, but also ecological, technological innovation and change, but also ecological and social innovation and change will be required to achieve that and I can go into those in more detail, but the other challenge that has to be seen against, in coordination with that is the dietary change. So we need, you know, so basically it's agriculture. You can't just look at it by itself.

**Reference 2 - 0.23% Coverage**

One is the degree of change and the risks around it, including like how the change was done and the potential unintended effects.

**Reference 3 - 1.24% Coverage**

I worry that the distinction made between transgenic. When it comes to a lot of like the issues around the current proposed regulatory framework the government has drafted, it's all around, it’s not necessarily around is it transgenic or not? And there's a kind of interesting question as to like how it, I think it's a simple way of talking to the public to say it's more risky to have genes from other species. If you're not doing that it's fine. I don't see that as a kind of black and white thing. I can see how if you had a gene from another species you're creating more unknown risk. But I'm kind of very interested in the bigger picture around risk. It can't be that simple, but it is that simple.

**Reference 4 - 0.42% Coverage**

So I don't have that kind of gut reaction of, you know, manipulating genes is immediately, you know, adding a gene from other species is immediately bad. I can kind of see how it would be that the risks aren't as simple as they are laid out.

**Reference 5 - 1.15% Coverage**

And I do it’s really, really interesting that the way gene editing is being marketed is very much simplistic ideas of risk, which I find really frustrating. I just wish there was, it’s such a polarised issue that there just doesn't seem to be any kind of really, the usual level of scientific discourse and debate around evidence and risks. It's just, it’s absent. It's just really frustrating. It seems to be either you can say it's wrong, it's risky, or you can say it's absolutely safe. This is in respect to my area of work and government policy. This isn't a reflection of like the academic world, but in terms of government advocacy and policy.

**Reference 6 - 1.68% Coverage**

Yeah, and a lot of people we work with in the kind of GM, GM world like GM campaigner world, they advise us on the fact that there are these unintended risks, and in some ways they can be, gene editing obviously allows you to try and target areas, but I've heard arguments that... I've heard all sorts of things. I've heard that by targeting a particular sequence of DNA code you're actually more likely to, if that code exists elsewhere, it's more likely to be in areas which code for genes. So therefore if you are doing, if you are doing a cut in a precise place and you accidentally cut other areas of the genome that you're more likely to disrupt genes and I mean, I'm not a geneticist. So like we hear these things, and then on the other side we'll hear that yes, unintended changes do happen but they're no different, no less risky, no less different than the kind of unintended effects that happen naturally through, you know, yeah reproduction.

**Reference 7 - 0.81% Coverage**

Yeah. So I mean, we hear lots of on different, this is all on the safety side. We hear lots and lots of different things and most like, from a personal perspective my concern is that it's always you hear, but it's risky from those who are clearly campaigning against it and you hear that it's absolutely fine from those who are campaigning for it and I just, I haven't seen the evidence really dealt with in a really, you know, neutral way, which is laying out…

**Reference 8 - 1.15% Coverage**

I mean one example of that is I've never seen any anything that would show exactly how both the intended change and unintended change of a particular gene editing event was equivalent in terms of risk as the changes that you could see naturally or through other techniques. Like it always seems to be, it's talked about but I've never seen like you know, evidence for it. I don't know how you would test it, but it's basically been described to me, the concept that something you can genetically engineer something to have changes that could have been done theoretically through other techniques or naturally, that's in the Bill.

**Reference 9 - 0.90% Coverage**

I presume there does need to be more research as always like there's no way we can say we understand genetics to the point that we can just not have risks, so that it's easy to say but we need more research, but I guess I'm more interested in like what actual, yeah what actually do you need in the regulation and just some really simple ones that strike me as sensible based on my current understanding of things like full genomic sequencing, and you know, some kind of analysis of those unintended effects.

**Reference 10 - 0.53% Coverage**

I feel like basically I want more evidence and more rational discussion of the evidence, like I see when I look in the medical context of gene editing it's very rational. You're allowed to talk about unintended effects and the risks of those and it's very much specific to a particular genetic event.

**Reference 11 - 0.42% Coverage**

then what happened was there was a lot of outrage from a lot of organisations about this because most people who are worried about GM are worried about the kind of, I guess the social environmental side of it rather than the safety side.

**Reference 12 - 0.54% Coverage**

But then the bigger thing is that the ownership of these companies, the way that they're being sold as solutions and it's just kind of unfortunately pushing out other solutions, a really simple one with you using crop rotations in order to manage weeds, you know, like really simple ecological solutions.

**Reference 13 - 1.48% Coverage**

You have to be rational. Always, always technology is pushing out other solutions just because of the psychology of our kind of techno-optimist minds. So like just stepping back and being really careful as to how it's used in practice isn't distracting from other necessarily innovation and solutions. So for example, the really basic one I mentioned before, which is like having good crop rotations in terms of potatoes, is a huge, huge, huge, huge thing, we really mustn't forget about, and diversity in the actual potatoes themselves. I mean the ideal would be that you end up with not just one type of potato that has this trait in, but you breed it into local varieties. So is the project going to look at that? Because that is so critical and that's the one thing I haven't mentioned is the implications in terms of diversity genetics.

**Reference 14 - 1.14% Coverage**

So why would that, and that's from a UK point of view. But assuming that things are going to change just because of the, you know, increased gene of it, I'm not saying, there may be good reasons why it will be different. But assuming it I'm not looking at that in detail as yeah, I think a really big risk. And yeah, so from the point of view of like potatoes and rice and things, for us it's, at the end of the day what you want is more local varieties, more local adaption and any kind of high, you know, high tech lab based approach to breeding has to integrate into that, and you have play on that, you can’t just assume it, you have to plan it.

**Reference 15 - 0.41% Coverage**

So I mean, I can't believe in a way I've left it out. My big passion is like we need more diversity in agriculture and there isn't, I've seen no reason why technologies like gene editing can't be part of that. Of course it could be.

**Reference 16 - 1.79% Coverage**

But I've never seen anything like really, other than, you know, advocates who like are trying to tackle that argument replying saying well, we'll allow you to use more, you know, you'll be able to do all this, but in terms of a plan of how that will happen. Yeah, and I mean that is my greatest worry, is basically we're just going to see a continuation of the patterns we've got, we’ve had inbreeding continue and accelerate potentially, when let's face it, we all want that increased diversity. So we all want those more local varieties to be, yeah, and I think it comes back to the fact that a lot of it requests social innovation, and ecological innovation, and that just gets underfunded. Lack of attention, and as a result, yeah, you end up with the one size fits all approach, even if you didn't mean to. So it’s depressing, but yeah, so I'm very much in the camp of yeah, more evidence, more of a science based. But there's no reason why these technologies couldn't be used in a way that would be beneficial.

**Reference 17 - 1.90% Coverage**

But the point, I thought I'd mention that at the end, because the point of that is very much to try and look at, take a step back a second from how you achieve, you know, what techniques are you using to achieve the outcome and actually looking at okay, hang on a sec, what is it that we want to achieve and looking at it through that bigger picture. Food systems lens of, and then like looking at plant breeding. So basically looking at like actually what do we actually need plant breeding to achieve? Like what's the actual vision? And then, you know, you start to bring into that the diversity bit, the local adaption side of it, the making, sure you’re not locking farmers into a particular reliance, and then you work back from there in terms of okay so therefore what are the breeding priorities? What are the blocks? What are the current issues? And that hopefully will be at least a tiny step towards addressing some of these issues in terms of like the lack of interest in anything other than yeah, I just hope that the high-tech solution will be a game changer.

**<Files\\Ginpu> - § 2 references coded [5.42% Coverage]**

**Reference 1 - 1.37% Coverage**

Yes, our membership definitely sees, and I see this as a solution, absolutely. And our lobbying, as you probably saw in that document, is very much framed around the climate and nature challenge.

[redacted reference]

**<Files\\Illabong> - § 1 reference coded [1.08% Coverage]**

**Reference 1 - 1.08% Coverage**

It's not needed, we don’t have this for conventional seeds so we don’t need this for the GMO seeds which in many cases are even safer than the conventional seeds from a biological perspective.

**<Files\\Jata> - § 5 references coded [10.23% Coverage]**

**Reference 1 - 1.24% Coverage**

I think for the European consumers, what actually was I think visible for them was the pandemic and the current geopolitical situation, I think these two are for the Europeans right now more visible from the perspective of the food security than the climate change.

**Reference 2 - 3.38% Coverage**

Well of course I think, first of all, let me say that I think that all different kinds of solutions should be used to solve these problems and one of them is biotechnology and I think the Europeans should have the same rights to use this science, this technology as all the other parts of the world, to be able to obtain crops which are more resilient to climate change which are more resilient to drugs, they could cultivate more crops in specific regions which would be more adaptable to those specific conditions and so forth or resistant to pests and well, I think we all know the examples, I don’t know how far I should go in that but obviously yes I do see the big role of biotechnology in facing these challenges.

**Reference 3 - 1.05% Coverage**

For the GMO’s I think we lost this battle so, at least what we try to do right now, I mean lost this battle against customers and politicians so, I think what we can do is to change the discourse of genome editing right now.

**Reference 4 - 3.05% Coverage**

Right so, I think first of all, it’s also important to understand the other stakeholders points of view and look for the similarities and if we compare science to the NGO’s, we all want biodiversity, we all want to eat healthy, that we spare the land, that we don’t use so much input, that we do not use pesticides and then build a kind of consensus around it and then communicate to the policymakers that there are certain goals and values that we all share and that there are certain ways to be able to satisfy these groups but not only to satisfy the groups itself but actually to bring the benefits towards the society and agriculture in the end.

**Reference 5 - 1.51% Coverage**

I think it’s coming back to this common value, what would you like to achieve within the agriculture in the coming years and how do you think yeah, what can you do to improve the agriculture because it will never be sustainable obviously, to be more sustainable and you will see that the values are the same, or similar.

**<Files\\Kebo> - § 5 references coded [10.67% Coverage]**

[redacted reference]

**Reference 2 - 1.82% Coverage**

RE So do you welcome the change in the UK then would you say?

PA It’s a start, a journey of 1,000 miles single step. I was hoping, especially in the light of the commentaries and also various bodies like the TIGRR Report… and also the Regulatory Horizons Council Report… are you familiar with that?

RE I am.

PA So that was also suggesting that there are certain specific examples where use of the GM method is clearly a good thing and barriers should be lifted promptly.

**Reference 3 - 0.78% Coverage**

I mean hundreds of thousands of tonnes of insecticides have not been applied thanks to Bt cotton, Bt maize, Bt eggplant etc. That’s hundreds of thousands of tonnes of neurotoxin have not gone into the environment thanks to this technology and they’re opposed to it, it’s completely insane.

**Reference 4 - 2.58% Coverage**

So one is that I have been wincing a lot over the years as people like George Eustace say no we don’t need GM anymore we’ve got this wonderful thing called gene editing and it’s going to solve all our problems, you know this is just scientifically illiterate thing to say, partly because the real benefits of gene editing are somewhat different than using the GM method but also I’ve a similar response to the response I had when people started talking about cisgenics rather than transgenics, and I squirm at the use of the word cisgenic as if it’s great, because it implies capitulating to the argument that there’s anything wrong with transgenics when there isn’t and of course politics is the art of the possible so that’s why I say I welcome the small step that was made in the DEFRA response making it easier to test gene editing outcomes in the field, I would say they should be doing the same for all of these, the GM as well as gene editing.

**Reference 5 - 4.15% Coverage**

[redacted reference]

**<Files\\Koshihikari> - § 14 references coded [17.71% Coverage]**

**Reference 1 - 1.94% Coverage**

No, I think there is plenty of stuff and I can send some stuff if you like. That says no actually, certainly for drought resistance conventional breeding’s much better. I mean it’s a simple thing that you know, the GM is you know, sort of tinkering around with one gene, maybe two genes. Whereas of course in that conventional breeding you can map on whole suites of genes. And so, when we come to things like drought tolerance then that’s you know, that’s multigenetic trait, so if you are tinkering around with one gene it’s definitely, it tends to be a very clumsy approach, just massively upregulating one gene. And we talk about increases in yield, marker assisted selection has you know, done some great things and it’s because it’s not sort one gene one function. It’s not one gene that sort, there’s no 1 gene that gives you drought resistance.

**Reference 2 - 1.24% Coverage**

And that, and many people, even Syngenta I’ve had conversation with, they say yeah you know conventional breeding is much better for things like drought tolerances, sort of complex trait’s that are controlled by more than 1 gene. So, they would tend to use Marker Assisted Selection or genomic selection. In a way you sort of, you’ve got your marker genes that tell you, oh ok the drought tolerance section of the genome, it may just be one it may be five.

RE Yeah.

PA It’s here and you can look in your offspring to see if you’ve got it.

**Reference 3 - 0.49% Coverage**

RE Process based. And why do you think that is?

PA I think because it’s this direct modification of the genome that can give rise to genetic errors. That can then result in unexpected and unpredictable effects.

**References 4-5 - 3.36% Coverage**

RE So do you think there is a route for approval of let’s say, a gene edited crop, can it ever satisfy enough criteria to be, to be made, well for the product to be in the market basically.

PA As an agricultural product, no. And I think there’s two things in there really, is, one is the deliberate environmental release. And then also the health aspect of consuming say the whole plant. Now I think that you could, for example, there’s one, like the fish oil in Camelina, do you know that one? It’s a UK thing, so you know… why don’t you just grow it in a greenhouse and extract the fish oil.

RE What did they say?

PA It wouldn’t be economic.

RE Wouldn’t be economic because of the size, do they need a field scale, ok.

PA So, you know that’s the sort of thing and I think it’s really, and I mean I would call that fish oil Camelina a pharmaceutical crop. Because it’s to produce a bioactive compound. But there’s no reason why you couldn’t do that in my book. I mean, hilarious originally, they were doing it in oilseed rape. DEFRA just said we are never going to let you have a field release of this. Because it would just so you know, contaminate everything.

RE Well yeah there’s grain all over the country.

PA Yeah, and you see oilseed rape… in Essex and stuff it grows at the side of the road, the middle of the road. You know so no, it’s not going to be contained in any, any respect.

**Reference 6 - 1.75% Coverage**

So, I don’t get it that all these things are going to happen, and we have already been uncovering, you know, with Rothamstead they’ve had these applications for various field trials. For various fancy things and none of them seem to work. We have this great press release about oh we are doing a field trial because of this; we are going to. And one of them may even be increase photosynthesis in wheat actually, and then everything goes quiet, no scientific papers are published, and you are like. Well, what happened to that one, and it didn’t work. But the PR was out there, oh we are doing a field trial on this and oh it’s going to be great, but it’s absolute horse shit. Just because you are doing a field doesn’t mean you are ready for a commercial application.

**Reference 7 - 0.21% Coverage**

RE So, do you think that the, in, the hype basically leads to broken promises?

PA Yeah.

**Reference 8 - 0.64% Coverage**

RE And then that maybe, that could diminish trust?

PA Yeah absolutely, absolutely. And I think you know once they are going to get, once they are going get caught, we have already had I think two, two field trials that have had really big ra ra ra and then they just died a death.

**Reference 9 - 0.51% Coverage**

As researchers I think understanding all the genetic errors. I think that’s got to be first and foremost. I mean it seems to me every week that a paper comes out about of target effect or what they call on target effects.

**Reference 10 - 0.68% Coverage**

You know the fact that damage is done as that sort of healing, and they have a DNA cut and that healing processes is done. So, you know that, that sort of thing needs to be understood and needs to be controlled. I think before you can really start to even think about any commercial applications.

**Reference 11 - 4.20% Coverage**

RE I did read, I did read a paper erm that, in fairness had intent a little while ago, that compared the amount of genetic, pfft, let’s call it add mixture, or actually I think they did refer to it as errors. That occur through, was it, a nuclear mutagenesis and then conventional breeding, GE and GMO and their claim was that there are more errors with conventional breeding or conventional sex than there are with GE. Do you think that’s…?

PA I’d like to see that expanded because I’ve heard that once or twice.

RE Right OK.

PA And it seems to me to not, any unexpected change is an error, well is that an error in conventional breeding? Is it part of the mapping process the same that goes on in animals? You know what’s an error?

RE Yeah yeah. And to be honest this conversation is probably more scientific than I thought it would go but…

PA Yeah.

RE As you said you think the most important thing here is to focus on, on or off target effects.

PA Yeah.

RE From gene editing which we don’t understand very well.

PA I mean, are they equivalent?

RE Right, sure.

PA Are the so-called errors in conventional breeding, are they the same, are they the same type, are they the same origin? Do they have the same effect? You know, nobody knows. For example, there’s this thing about, the one you’re doing say, gene editing, you can access any part of the genome. Whereas in fact you can’t with conventional breeding, some areas are, it’s all to do with the way the DNA is coiled, so some areas are accessible, some areas aren’t accessible. So, in some ways that’s limited then, those, any sort of changes is limited. Now does a change that’s within that inaccessible area to conventional breeding, if that’s accidentally changed by gene editing, is that an error that wouldn’t occur in conventional breeding.

**Reference 12 - 1.13% Coverage**

And then, a knock-on effect. You know that gene might have had another function elsewhere. So, these are the sorts of things that need to be investigated. You know it’s quite easy to say look, we’ve done this, we’ve tweaked one gene, that’s all it is. But actually, you don’t know the Implications of that. And that’s really where the questions lie, where the sort of research needs to be done. The other thing with that, we’ve only tweaked one gene, was that we had a look at DuPonts Waxy Core.

**Reference 13 - 1.10% Coverage**

So, they introduced six plasmids in there, so say if you look at Roundup Ready Soya or BT corn that’s one plasma, that’s one GM insert, they used six. Every Time you put in a GM insert, there’s rearrangements and deletions of the host DNA. So, you may have only tweaked on gene, but you have inserted 6 inserts and supposedly got rid them of them. So, hang on a minute, is there any affects from that? You know gone are the days where you say, well it still looks like a Soya bean.

**Reference 14 - 0.47% Coverage**

… and a classic is you know, you know, don’t you want a GMO that can make your hair grow back if you’ve gone bald, you know all these ridiculous claims. And that’s like, that’s not what’s on the table.

**<Files\\Matla> - § 8 references coded [11.42% Coverage]**

**Reference 1 - 1.71% Coverage**

We ask more and more of crops, they need to be good yields and to be good for technology aspect such as, just like oil for instance, oil quality or sugar productivity for sugar beet but we start for twenty years basically to ask also performance regarding plant resistance. So, we ask a lot and for sure, the way farmers grow crops are really important, but the potential is the variety – you also know that for decades we have had a lot of tremendous success increasing yields, increasing quality and also increasing resistance to some pests but NGT could be really a tool to help to boost these criteria.

**Reference 2 - 0.84% Coverage**

I mean you know; it is easy to do a cross, but it takes many years for a variety that you can put on the market. The idea is that with NGT you can use this time especially focussing for one mutation that could be improved and putting in your varieties that are already suitable for the production.

**Reference 3 - 1.24% Coverage**

So yes, just to sum up, the [association] is really in favour of that because we see, we hope it can be, you know it has just opened a new reasons- a lot of promises, we’ll see. I don’t want to be the one telling that it will be the key for the future, it is just a tool because I spent so many years at the bench, I know that sometimes you think something could be useful but in the end it is not – it is just potential, it gives you hope.

**Reference 4 - 0.41% Coverage**

… so with NGT again you can really easily see the hope breeders can have is that just faster everything, all the selection of the breeding process.

**Reference 5 - 2.32% Coverage**

… it is really key a good question because also with NBT you can do a lot of things, I mean it is not for nothing that they got the Nobel prize, it is just a revolution even for a lab., when I was in [institute] it was the beginning of that- well, Arabadopsis naturally because you already had a lot of tools, but working on crops they start to get really exciting about this CRISPR, finally they can do what they want and very precise, accurate tool to modify one mutation that you know that could give a result so, for breeders it is just also amazing because again it gives hope, as long as you know the gene, this is the first step, you need to have a lot of research to know, to identify which allele could have the potential to help you to reach the goal you have set for the yield or for crop resistance for instance.

**Reference 6 - 1.73% Coverage**

So, that is the difference between directive and regulation. I don’t know if the Commission would like to go to have a discussion, have a new regulation, it would be a very long process, we need to have access to NBT fast, if we wait 10 years because we need to have this 10 year long debate, discussion about how we renew the directive, we lost the race against China or United States or even South America or Africa, because they are our opposition and they can basically, or UK also, because they are already in favour. They see the potential and they can go a bit faster than us to change their regulation.

**Reference 7 - 1.99% Coverage**

So, I mean, it is fair that the society takes an interest in the topic, they do not really have the key to really understand what is going on and if you speak about a new technique, they are a bit afraid, you can see that with the vaccine against COVID, everybody is afraid but they don’t know what is RNA, they don’t know what a PCR – every day they speak about PCR and everyday and they don’t know, maybe those vaccines can be natural some not. I mean everybody would like to be an expert, even if they don’t know and so, you use the new technique that can edit genome so I can understand that you have some discussion, society would like to have a discussion, evolution process would like to make sure.

**Reference 8 - 1.18% Coverage**

… it is very easy for the NGO to say look, this is a new GMO or hidden GMO. This is how people call NBT in France, they call them the new GMO or the hidden GMO. It would take years to convince those people that it is not exactly the same as putting a transgene from another plant or other bacteria into your plants because here you are just using a tool to create a mutation that you know that can improve some qualities.

**<Files\\Njavara> - § 8 references coded [13.49% Coverage]**

**Reference 1 - 0.30% Coverage**

It would not make any sense, huh? You want to improve nature so how could it be nature identical or something like that?

**Reference 2 - 3.39% Coverage**

So, this reminds me really of what I learned at university 20 years ago. You know, what has been discussed earlier, that at that time everybody was saying, “Ah, now we can sequ-, it’s only a few years and then we can sequence the genomes like we want to and you can read it and then let us know. Like in an open book. Then we can divide plants however we want. Then we have the gene for here. Then we have the gene for stress-tolerance and things like that and we can easily transfer them from here to here.” And you know what came out of it, yeah? Because these traits are so complex. And they are not only based on genetics. So, that’s-, I think this hasn’t changed for the new techniques in the end. They can do everything easier and more precise. Yeah, that might be true. But the whole genetic basis of what’s been done hasn’t changed. We’re still talking about complex genes. We’re talking about what’s, let’s say, alternative splicing. We’re talking about epigenetics. About the silent regions in the genome which used to be called junk DNA and-,

RE Yes.

PA Now we have identified that they maybe still play a role in adaption to rapidly changing environmental conditions in the end. So. That totally makes sense, yes? So, why should evolution copy large regions of the genome in every cell cycle without having a use for it? So, that would have been totally a waste and, I doubt that this would have survived evolution.

**Reference 3 - 0.58% Coverage**

I think if the big promises would be fulfilled, then it would be very easy to go to any regulation process to any authorisation. Everybody would dare to use these plants. So, let’s go for it. Let’s just prove me wrong. Please, prove me wrong. Yeah.

**Reference 4 - 1.18% Coverage**

I’m really wondering about that because this is what evolution has worked on for millions of years and it’s the enzyme that’s-, which is the most-, can be found in every plant all around the world. And every plant’s seeking to improve its photosynthesis in the end and every mutation that would improve photosynthesis would make its way. And so, I’m really curious to see how the approach would be. And, again, I know the approaches because I’ve seen a documentary on that. Some days ago, I think, but-

**Reference 5 - 1.28% Coverage**

RE Mm. So, coming back to something you were saying about the sort of questions you had around photosynthesis, what do you think to the idea of improving the productivity of crops, for example? As an overall aim in plant-breeding? Is that the right approach?

PA I think it’s one approach that we-, and of course, it should be followed. There’s some productivity and-, but I think we’ve, in the past, the focus has mainly been on productivity, in the end, and maybe not so much on other traits, yeah, which we desperately need in the end.

**Reference 6 - 2.21% Coverage**

I can see why [inaudible] though when you see these-, all this Corona crisis. What states can move and what they are willing to invest for such a crisis. Yeah, that’s something different, maybe. But when it comes to breeding, this is what makes other breeding techniques so much more attractive to me than genetic engineering because then there you can follow more traits at the same time, yeah? And I think this has been not really reflected in breeding in the past. It’s more or less focused on yields, or on high yields or on protein quality of the-, things like that. But not on a tolerance to antibiotic stress or for the needs that organic farming has and things like that. And, on the other hand, we have made some big achievements with biotechnology in the end, when it comes to molecular markers and genetic markers. But it’s not GE, yeah? And I think this is a technique that we are very much in favour of.

**Reference 7 - 1.91% Coverage**

RE I mean. I know that the, you know, the crop scientists on our project will and there’s probably, down the line, there will be some attempts at commercialisation, but for me and my supervisor, at this point, we’re really much more about understanding the societal aspects of this proposition. And particularly, at the moment, just really around governance of biotechnology crops. You know, what does effective and good governance look like in this space of these crops?

PA Yeah. I would maybe work on the whole framing because I think when you make such big promises to improve photosynthesis, this is a very-, it’s a very big promise. Very. It can easily be challenged. The design.

**Reference 8 - 2.63% Coverage**

PA Maybe rice is, of course, more interesting as a-, but, you know, what we’ve seen in the past is that there have been a lot of projects where some achievements have been made with genetic engineering. Yeah? But they never make it out of the lab. That’s really-, financially, you have this basic research. And it’s not for regulatory reasons but it simply doesn’t work on the field. Or already on a bigger scale in a greenhouse or something.

RE Yeah. As you say. No. Something I’ve realised is that there’s a bit of a trap for the proponents of these technologies. Because if they make big promises, it’ll be held against them. But if they-, they also want funding. So, they have to say, “Here’s what we could do. In theory.” But if they struggle to achieve those things, because they hyped the thing too much-

PA Yeah.

RE Then it’ll be held against them and-

PA But I saw an analysis in [location] of where the funds-, the public funds go to, and this is not going to organic farming, or something. It’s always going to see to such projects, which are really using these high technologies, in the end.

**<Files\\Ofada> - § 2 references coded [2.77% Coverage]**

**Reference 1 - 1.64% Coverage**

Yes, I think the political justification also for the Commission is exactly this positive mentioning of the biotechnologies to contribute to these policy objectives, which was also confirmed by a number of scientific studies of course that already showed that these type of technologies with certain products can have beneficial impact on reducing pesticide use, fertiliser use, adaptation to climate change etcetera.

**Reference 2 - 1.13% Coverage**

I mean if you develop material and you can exchange material or not really do field trials, so I think it’s also a disadvantage for European researchers and so trying to align let’s say, policy approaches around the world in the sense, it makes sense for all different kind of people.

**<Files\\Ondel> - § 5 references coded [16.88% Coverage]**

**References 1-2 - 3.90% Coverage**

RE So the NGT file lands on your desk. What are your first impressions of that?

PA Well, I mean, I think like any person, any other person like when I saw genomic it felt kind of a bit kind of scary.

RE Yes.

PA It's all associations with GMO but actually once you start digging in and trying to understand the actual of the idea then you understand that it's actually nothing to do with GMO and also from what you hear, because [personal information] they talk a lot about transforming how European agriculture should be working future, a more sustainable way and they're, and NGTs is offered as one of the actual solutions to facilitate this transformation of European agriculture to make it more sustainable. Rely, reduce reliance on different chemicals and I have a positive look at it and, but of course I need to have more understanding of it.

**Reference 3 - 0.44% Coverage**

And that's what we're all doing in our organisation. We're trying to understand it and form our opinion.

[redacted reference]

[redacted reference]

**<Files\\Patna> - § 21 references coded [25.40% Coverage]**

**Reference 1 - 1.02% Coverage**

But I also think that a lot of it is just the mentality, the way of approaching it, the thinking that gives us genetic engineering in crops is the same thinking that gives us monocultures and it gives us corporate control because if there’s this concept that there is a perfect wheat, there is a, you know there is a best potato and if we can just make the very best potato then everything will be brilliant. Of course, the biggest problem is having one kind of potato, you know, resilience relies on diversity. Environmental resilience and economic resilience relies on diversity.

[redacted reference]

**References 3-4 - 0.82% Coverage**

RE Do you think there’s a way in which, for arguments sake, gene editing could be in public hands and could be used to enhance diversity as opposed to how it is used now to kind of further monocultural industrial farming or do you think that it’s a lost cause, do you think that the technology itself is a lost cause because it has these inbuilt properties.

PA So I think the, the technology’s inherently risky and much more complicated than the PR is telling us.

**Reference 5 - 1.63% Coverage**

So assuming we’re going to come back to that side of it, then my personal view is that you can’t apply an ethical bar or moral kind of judgment on any technology. There are others who disagree. There are certainly [organisation] members and individual supporters who just think it’s wrong. They just don’t, you know, they’re kind of playing God arguments for something that is actually, you know a kind of religious spiritual position and for others, I think it is, there’s a really interesting thing that people who don’t necessarily have a lot of scientific vocabulary will often express things in those terms. They might say oh you know, it’s like playing God and actually, if you can spend time with them and really delve down then what you get to, is law of unintended consequences and that the public actually has a really strong understanding that when you mess with things you don’t understand, really bad stuff can happen.

**Reference 6 - 0.74% Coverage**

I mean that was mentioned in almost all of the studies that people just went well, what could happen, you know, we don’t know.

PA Yeah, what don’t we know. I just never quite understood why Donald Rumsfeld got such a hard time because he’s so, he’s so right.

RE This is for other interviews yeah, Rumsfeld, he was trying to say something very astute but.

PA Incredibly important, yeah, you know. Unknown, unknown.

**References 7-8 - 2.01% Coverage**

RE To throw this back though, that came up in another interview with a plant scientist who did say that we have had GM for 30 years and we know that that can be applied safely, I use that term advisedly because… I think Glyphosate is probably the bigger issue with that first generation GM because of the package it was supplied with let’s say. But GM, yeah, a transgenic event. Let’s say one transgenic event. He would argue and he did, that we’ve had that for close to 30 years. That itself is reasonably safe.

PA See I just find that such an extraordinary statement for a scientist to make that like we’ve had, we’ve got two types of GM growing at any scale whatsoever. We’ve got weedkiller linked crops and we’ve got insect killing crops. Two things in 30 years. So like, at virtually none of the promises that were made in the early days have actually come to fruition. So you know, that’s this and you’ve got also, what kind of a scientist looks at two examples of the application of a technology and goes, oh, the technology’s fine. We did two things with it and nobody’s found a specific problem with it.

**References 9-10 - 1.40% Coverage**

So, actually it’s a really, really complex process and I think, I don’t think you can say that any of these processes are inherently safe or unsafe. They’re, it’s a set of techniques that can go wrong. It’s a set of techniques that have inherent risks and the idea that you could say it is or it isn’t safe is extraordinary. I think you can probably say, if people say it’s not safe meaning, we don’t understand enough about the risks, we can’t proceed, I’m not happy to proceed, I don’t believe we know enough about the risks to mitigate them properly but, it, the other thing going back to the GM that we have, well, actually, who’s studied what impacts it’s had on the population? There have been no population studies of those who have eaten GM versus those who haven’t. So how would we even know?

**Reference 11 - 1.54% Coverage**

It’s not in the, the biggest sort of dietary study that I know is the Epic which is focused on cancer, but it’s run in Europe so you won’t have a large population that has eaten it and I’m not suggesting that there is a thing or there isn’t a thing, but I just find it extraordinary that anyone who thinks of themselves as a scientist would say, it’s all fine when we haven’t been looking at it. You know, it all seemed fine, feeding herbivorous animals, animal flesh until BSE happened. Like the thing doesn’t go wrong until the thing goes wrong. And if you’re not looking, you won’t know but, I mean absolutely that you know, the environmental impacts are by far the biggest thing that’s happened. You know the impact on monarch butterflies and biodiversity more generally, plus, oh guess what, you know, the weird evolved resistant. Who would have imagined that evolution would.

**Reference 12 - 0.21% Coverage**

Well maybe they will but they haven’t done it yet have they? They haven’t got anything that replaces neonicotinoids.

**Reference 13 - 2.43% Coverage**

Presumably. I think the big thing, you know, what I would say here is that, well first of all, it brings up the [unclear]. You know, so once we’re using that technology we’re getting into a different field, why would it last, what it actually is what are they going to do, not just going to trigger more evolution. If this is about pest management then, do you know, you really, it’s the same mentality. Instead of spraying a chemical, it’s changing the molecular structure of the crop, it’s still thinking let’s you know, let’s get, let’s beat these [unclear] at a molecular level. Whereas, you know with the integrated pest management, you’re kind of working with nature. So I still think it’s still seeing nature as the enemy rather than a tool that we can use. It’s still trying to dominate and so there’s still that kind of quite troublesome thinking. But it’s also, it’s not remotely a reason to deregulate. I find, this stuff comes up all the time. It’s kind of, we’re the ones who are going to harm the environment because we want there to be regulation of genome editing and genome editing could do all these marvellous things. Well GM was supposed to do all those things and it didn’t, so I am quite cynical about what it actually will do. But the use of hypothetical potential as a reason why we should allow free rein is just extraordinary. There’s a huge leap there of logic.

**Reference 14 - 1.85% Coverage**

RE Can I assume also that you don’t think there should be a difference made between like a transgenic crop or what we’re calling a cisgenic crop?

PA Yeah absolutely not. These divisions are completely artificial, and you know, like, gene editing is GM with better PR, there is absolutely nothing in any of the legal documentation that ever said it was about transgenesis. Some early campaigners, I mean I wasn’t, I’ve been doing this job for about seven years, so I wasn’t around you know, in the early days. But lots of the campaigners did this whole kind of, oh my god, they’re putting fish genes in a tomato. It’s just a campaigning thing. That doesn’t mean that that was the only thing that was a problem, it just means that that was a thing that people could grasp and it was sort of entry level discussion and there has been an absolutely concerted PR effort to distance new techniques from older techniques and there’s some stuff said that is just simply untrue by government ministers, by you know, less so by scientists, it’s quite interesting.

**References 15-16 - 1.61% Coverage**

But I actually think, what I would love to know is whether a lot of the people who see that genuinely picture in their head that genome editing is like some kind of microsurgery where they’ve got such a good microscope.

RE Like take it apart, put a bit in, join it up.

PA Yes and if you actually showed them that what you’re doing is putting chemicals in a, you know a cocktail of things in a test tube and putting it through a centrifuge and hoping that it goes in the right place, they would see it very differently and you know, when GM first started, that was supposedly really precise and targeted and just going to do, you know, was going to change things in a brilliant way. Nobody ever talked at that time about the fact that they had no idea where the DNA they were adding ended up on the genome.

RE Where that would be expressed, yeah that’s true.

PA They had no concept and they never admitted that.

**Reference 17 - 2.49% Coverage**

But you see, what I’d be interested to know if you’ve got from that and it may not have been where we’re heading with the questions but, like we’re often sort of confronted with this, you know, like it’s happening in medicines. You’ve had the vaccine. But how can you possibly be against it, but, treating, like every treatment you have, whether it’s prophylactic or you know, treatment of a condition you already have, you’re making a risk benefit analysis on an individual basis. So, you know, you consider, you might do it very quickly, but you know, you consider whether or not, you know, if you’re tired, you consider whether or not to drink coffee on the basis of whether or not it will keep you awake later. All this kind of stuff. People make that calculation very rapidly. You can’t do that with your food if it’s, you know, if it’s just out in the environment in an uncontrolled fashion. So I think there’s the uncontrolled nature of it, once it’s out in the open environment. But also, you know, medicines are prescribed for people who have something wrong with them. Food is eaten by every single person, at least three times a day in some of our cases, seven or eight you know. Like, we, it’s a fundamental part of what we do all the time so, actually, you can’t apply the same framework. Plus the regulations on the uses in medicine are much tighter. You know no one’s trying to deregulate in medicine are they?

[redacted reference]

**Reference 19 - 2.52% Coverage**

See because it, I just think there’s two huge issues that are completely missed in that. I mean one is that you know, what’s the problem you’re trying to solve. If it’s world hunger, people are not starving because photosynthesis is a bit rubbish. People are starving because they are poor and they’re not going to stop being poor because you’ve slightly improved the photosynthesis in their rice. They won’t have any more control, they won’t have anymore access to land, anymore food sovereignty so I just kind of like, it feels like you’re looking at the wrong question and I think, the what I fear happens, and this is partly about the way our education system works, that people specialise so young and so early in life, is that really, you know, good people who want to do good in the world and who have spent the first ten years of their career doing genetic biology, ask themselves how can I use this particular form of extreme cleverness that I have developed in myself to do a good thing. Oh I know, I can make photosynthesis a bit better. But like actually what we all need to ask is what’s the problem, what’s the real problem. The problem is not photosynthesis, I mean really photosynthesis is done quite well so far, hasn’t it. And maybe, you know there is almost certainly a reason why, like there’s a payoff. There’s a payoff in the plants that have, you know they might be more efficient to photosynthesis but what else is there.

**Reference 20 - 0.62% Coverage**

Yeah absolutely. It’s access, it’s waste, it’s you know, control.

**Reference 21 - 1.03% Coverage**

But you know, imagine if that level of, like it’s not like people were born good at genetics you know. If they had had a broader education and they’d actually, you know, gone down, if engineering didn’t have lower social status and they’d gone down that route, they could be solving waste you know. They could be looking at the transport issues. It’s not like everyone’s going to go into politics and deal with that side of it although we do need, you know, people with a broader education in politics. But there are other, actually technological problems that could really do with solving.

**<Files\\Rufipogon> - § 9 references coded [12.44% Coverage]**

**References 1-2 - 3.37% Coverage**

PA Of course, yes, well the GM rice, I think you mentioned GM rice so the GM rice has been developed in the Philippines so there’s a letter a couple of years ago, 2018 something like that which from the Nobel Laureates, which accused Greenpeace of crimes against humanity on the basis of their opposition to the GM rice and that was a complete joke. That was 100 people, Nobel laureates who have no expertise with GM rice really misusing their authority as they’re claiming an authority over the topic on the basis of their expertise in another space in another field and then accusing Greenpeace of crimes against humanity which is, that’s not the issue. But the issue is that they based this, they said they don’t know what they’re talking about because the science says that it’s safe. So you know, it just says that the experts that want to keep this science base and exclude others just don’t even understand the arguments being put forward by the opposition.

RE I see.

PA Greenpeace and others aren’t making the claim that it’s not safe. That’s one of their claims. That’s not their only claim. The majority of the claims in opposition to GM crops are corporate control. There are ethics around naturaleness which are really difficult to solve. Corporate is the one we could address right. The third one is they definitely do what it says on the tin, right. It says it’s going to feed the world. That’s BS to be honest, this is not going to happen.

**Reference 3 - 2.47% Coverage**

We’ve known this since the late 1990s and there’s more and more public engagement, I mean I see this like. I’ve just been asked to be an expert witness on another citizens forum for plant biotech where they’re going to do some kind of health [unclear] which is going to be some kind of you know, there’s just no point really. I can tell it’s a waste of everybody’s time. We know what’s going to be said. We’ve done enough public surveys, public focus groups, citizen juries, public dialogues on GM and genome editing to know exactly what the public are concerned about. What we lack is action on that and what we lack is experts listening to that. Your point earlier about you know, response renovation, scholars and social scientists that come into disciplinary and presenting at conferences, we were just talking about the conference we were going to present it to the chemical industry rather than talking to other social scientists because it’s harder that way. Part of the experts’ failure to understand what the public are concerned about is in part social scientists failure to get that across.

**Reference 4 - 1.47% Coverage**

… the other thing that people are concerned about is that GM crops don’t solve the problem, they don’t solve anything of value to the public. The public don’t care about whether Monsanto’s profits increased by so much or whether farmers planting soya beans over thousands of hectares in the US saved some time in their weed killer applications. They care about children in the Philippines with eye disorders because they don’t have access to good vitamins. But even in that case, a lot of the public are sceptical about in a sense that none of the causes of vitamin A deficiency is poverty. And you can try and solve it with changing rice to incorporate…

**Reference 5 - 0.32% Coverage**

PA So when you said tell me about the project.

RE Why photosynthesis?

PA Well yes, surely like the plants have mastered it themselves.

**Reference 6 - 1.12% Coverage**

PA Who are they imagining is using this?

RE Oh I see what you mean.

PA Who are they doing this for?

RE Well, farmers in places where they might get hotter.

PA Which is where?

RE Is their argument, I guess it’s all over at this point.

PA Well you see, that for me is an immediate non starter. So unless they’ve done the thinking around who that is, I want to know who, I want to go and meet them and so they’ve been to that place and understand this is where it will be grown.

**Reference 7 - 1.99% Coverage**

PA But again productivity is such a …. what does that mean. That is somebody producing it, somebody producing the potatoes and you would see an increase in productivity in that, so where is that, where do they think that’s going to happen, which farms?

RE I assume northern Europe, so whoever would buy the eventual product.

PA But I guess I would want to be pushing that hard. I would want to see them on the ground, so this is one of the failures of the GM Diamondbacked moth in New York.

RE Well my job is to go out to the farmers and actually find out where’s the demand but I know what you’re saying, it should have been done prior…

PA Not even the demand. It’s about whether the… what is the reality of their situation. What is that local context of farming potatoes in northern Europe in a particular place. What does that look like. Is productivity a problem.

**Reference 8 - 1.11% Coverage**

Because we have the same reason I just told you about the [company] case, we’re world leaders in the science around this and we can’t plant growing crops it makes us look like idiots. And we’re not capitalising on the economic potential of economic growth from this. Whether it actually solves real problems or not I don’t know. I mean does the project you’re working on, do you think it actually solves real problems, or do you think it’s still very speculative and a hammer looking for nails.

**Reference 9 - 0.60% Coverage**

When we look at these projects that’s what we see right. And feeding the world, it’s a non starter, like we know that we can feed the world right now. Feeding the word is not a technical problem, it’s a political problem. So I think that’s where the charge will be.

**<Files\\Salari> - § 3 references coded [7.51% Coverage]**

**Reference 1 - 2.08% Coverage**

The [movement] has been historically opposed to genetic engineering in farming. This started around the 1890s when it came up, when there were also big promises made, big expectations also regarding rice that has transgenic genes in it to produce vitamins, for example, which then led to a situation in which those benefits did not materialise as promised. This was kind of also a reinforcement for the organic movement of look, there were these big promises of pesticide reduction, of solving world hunger, and these benefits have not materialised in the end.

**Reference 2 - 2.05% Coverage**

But we have seen a lot where these techniques are being used to introduce transgenes for pesticides resistance or introducing plants that produce the toxins themselves, like the Bt plants. So there was a big reinforcement of any [people] that this was the right decision to not go this way of looking at this very narrow scope to isolate plants to single genes or multiple genes. So to go away from this kind of narrow fixes but more towards focusing on agricultural processes, like inter cropping, for example, crop rotation, to approach it from this.

**Reference 3 - 3.38% Coverage**

I mean, it has been clearly stated, and then a bit washed down, that these new genomic techniques will reduce pesticide use and increase resilience towards climate change and related weather conditions. Even though, if you look at the evidence, well there is not much evidence. There is evidence on yes, there are some genes that maybe are involved in drug tolerance and you could change them. There is not much further practise or like experience from the field that does prove these kind of claims that go very far. It's a big statement to say that these genomic techniques will save us from climate change and reduce pesticide use, and the [movement] would have liked that these questions are treated more with an open mind, based on the current evidence also, and to clearly indicate if something is maybe hypothetical at the moment and might realise in the future, but it's not necessarily ready at the moment.
